# LncRNA EP300-AS1 interacts with PTBP1 to destabilize PRMT5 mRNA and suppresses NSCLC growth and metastasis

**DOI:** 10.1038/s41419-025-07931-3

**Published:** 2025-08-11

**Authors:** Jingyi Chen, Chongyu Fan, Songze Song, Deyu Zhang

**Affiliations:** 1 Department of Gastroenterology, General Hospital of Northern Theater Command, Shenyang, China; 2 Department of Thoracic Surgery, Shengjing Hospital of China Medical University, Shenyang, China; 3 Postgraduate College, China Medical University, Shenyang, China; 4 Department of Cell Biology, Key Laboratory of Cell Biology of National Health Commission of the PRC, China Medical University, Shenyang, China

**Keywords:** Cancer, Diagnostic markers

## Abstract

Non-small-cell lung cancer (NSCLC) is one of the most common types of malignant cancer, characterized by high rates of metastasis and mortality. However, the molecular mechanisms underlying NSCLC growth and progression remain largely unclear. Here, EP300-AS1 is identified as a critical tumor-suppressive long non-coding RNA (lncRNA) in NSCLC. EP300-AS1 inhibits NSCLC cell growth and metastasis both in vitro and in vivo, and is associated with better clinical outcomes. The function of EP300-AS1 depends on EP300-AS1-PTBP1 interaction and PTBP1-mediated PRMT5 mRNA stability. EP300-AS1 binds directly to PTBP1, preventing its cytoplasmic translocation and PTBP1-PRMT5 mRNA complex formation in NSCLC. In the absence of PTBP1 binding to the PRMT5 mRNA 3’-UTR, PRMT5 mRNA stability and expression are reduced. PTBP1 knockdown or PRMT5 inhibition abolishes EP300-AS1-regulated NSCLC cell proliferation, migration, and invasion. In patients with lung adenocarcinoma (LUAD) and lung squamous cell carcinoma (LUSC), EP300-AS1 expression is negatively correlated with PRMT5 expression. Overall, these findings establish the EP300-AS1-PTBP1-PRMT5 axis as a key regulatory pathway in NSCLC progression, providing a novel regulatory mechanism and a promising target for NSCLC prediction and therapy.

## Introduction

Non-small-cell lung cancer (NSCLC) accounts for more than 85% of lung cancer cases and remains the leading cause of cancer-related death worldwide [1]. Despite advances in therapy, metastasis in NSCLC, mainly including lung adenocarcinoma (LUAD) and lung squamous cell carcinoma (LUSC) subtypes, usually leads to treatment failure. Molecules involved in NSCLC metastasis include growth factors, adhesion molecules, chemokines, and complex gene networks [2]. However, due to largely unknown molecular mechanisms and limited therapeutic options, it is important to explore novel targets and mechanisms for NSCLC treatment.

Non-coding RNAs (ncRNAs), which do not encode proteins, were long considered “junk sequences”. Recent research has shown that tens of thousands of ncRNAs, including microRNAs (miRNAs), circular RNAs (circRNAs) and long non-coding RNAs (lncRNAs), regulate tumor growth and metastasis, revolutionizing this field [3]. Representing more than 90% of transcribed RNAs in the human genome, most ncRNAs remain poorly characterized [4, 5]. Unlike small ncRNAs, lncRNAs are transcripts longer than 200 nucleotides and typically possess a 5’ cap and a 3’ poly(A) tail [6]. Studies have shown that lncRNAs can regulate cancer-related gene expression through various mechanisms, such as modulating transcription of nearby genes, acting as miRNA sponges, or binding to proteins [7, 8]. As the first lncRNA identified, metastasis-associated lung adenocarcinoma transcript 1 (MALAT1) has been recognized as a key regulator of lung cancer metastasis [9]. MALAT1 acts as a sponge for miRNAs including miR-339-5p [10], miR-144-3p [11], miR-22 [12], miR-625-3p [13], and miR-338-3p [14]. In addition to interaction with RNA, MALAT1 also binds to proteins. For example, it facilitates the localization of nucleolin and nucleophosmin to nuclear speckles through direct interaction [15]. MALAT1 also binds to heat shock protein 90 (HSP90) and reduces its ubiquitination, leading to HSP90 protein stabilization [16]. MYC-induced lncRNA HIF1α-AS2 has been shown to epigenetically activate MYC transcription through a positive feedback loop, promoting KRAS-driven NSCLC cell proliferation and metastasis [17]. Another lncRNA, RMRP, enhances transforming growth factor beta receptor 1 (TGFBR1) transcription by recruiting Y-box-binding protein 1 (YBX1) to the TGFBR1 promoter, thereby promoting NSCLC growth and progression [18].

In this study, we identified four differentially expressed lncRNAs associated with NSCLC growth and metastasis through bioinformatics analysis, including MIR210HG, LINC01116, SFTA1P, and EP300-AS1. Previous studies reported that MIR210HG promoted proliferation and invasion of NSCLC through the DNMT1/CACNA2D2 and DNMT1/SH3GL3 pathways [19–21]. LINC01116 drove oncogenic phenotypes in NSCLC by activating RNA polymerase I transcription [22], reducing IFI44 expression [23], and targeting the miR-9-5p/CCNE1 axis [24]. LncRNA SFTA1P activated the LATS1/YAP pathway and inhibited NSCLC cell proliferation and metastasis [25, 26]. However, EP300-AS1 was mentioned as a potential prognostic factor in LUAD models through basic bioinformatics analyses [27, 28], but its comprehensive functions and integrated molecular mechanisms remained largely unknown. This study further examined the biological function and molecular mechanism of EP300-AS1 during NSCLC progression. Our study found that EP300-AS1 suppressed NSCLC cell proliferation, migration, invasion, and metastasis in vitro and in vivo. Mechanistically, EP300-AS1 directly interacted with the RNA-binding protein PTBP1. This interaction not only prevented PTBP1 cytoplasmic localization, but also blocked PTBP1-PRMT5 mRNA complex formation. Without PTBP1 binding to the 3’-UTR, PRMT5 mRNA stability and expression was decreased. Collectively, this study demonstrated that the EP300-AS1-PTBP1-PRMT5 axis acts as a novel pathway involved in NSCLC progression.

## Results

### EP300-AS1 is screened out as metastasis-related lncRNA and associated with NSCLC clinical outcome

**Fig. 1:**
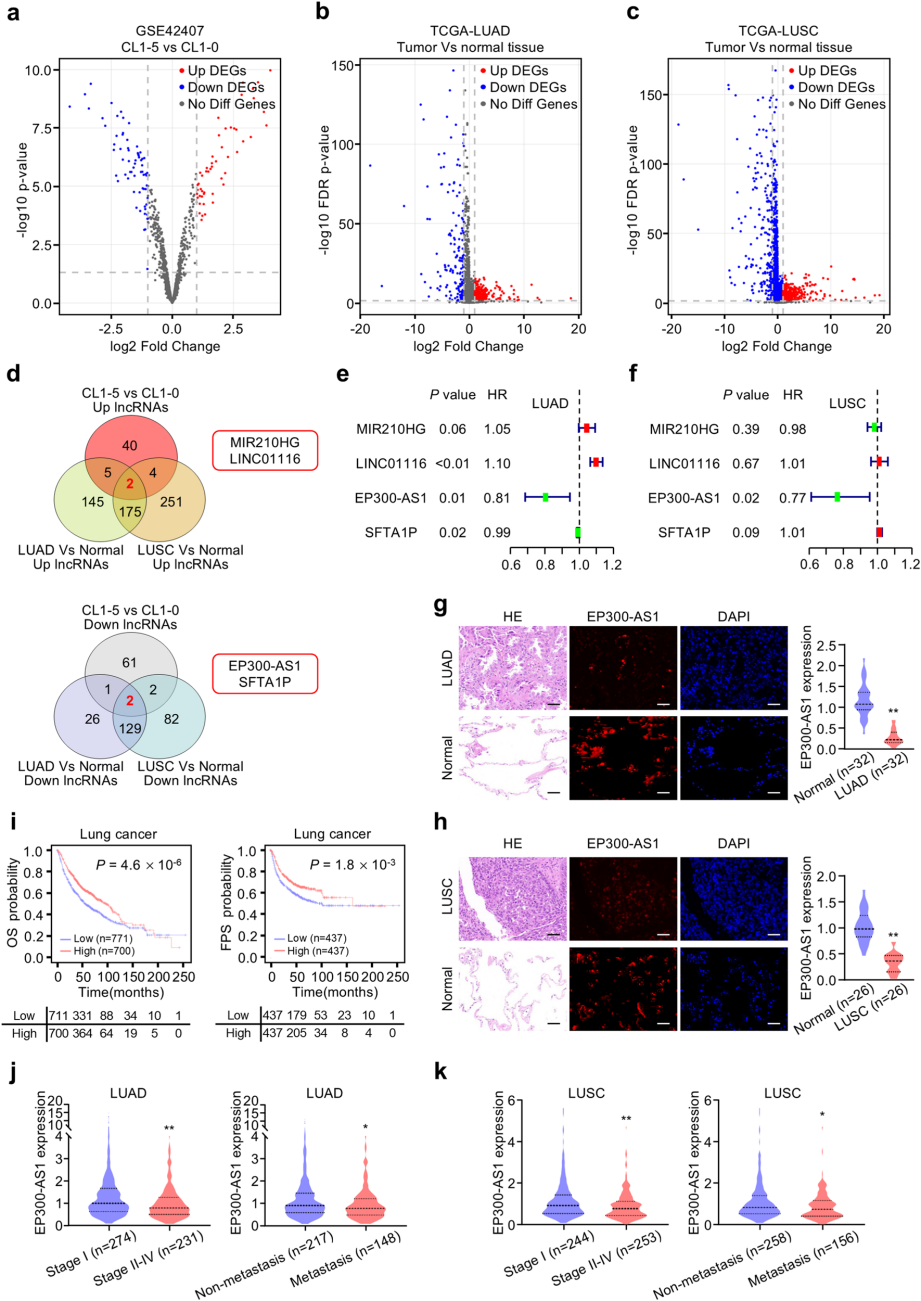
Identification of EP300-AS1 as metastasis-related lncRNA in NSCLC patients. **a** The volcano plot showed differentially expressed lncRNAs between high metastatic CL1-5 cells and low metastatic CL1-0 cells. **b**, **c** The volcano plot showed differentially expressed lncRNAs between TCGA-LUAD tissues (**b**) or TCGA-LUSC tissues (**c**) and normal tissues. **d** The Venn diagrams were used to identify four differentially expressed lncRNAs as metastasis-related lncRNAs. **e**, **f** The forest plot depicted a significant negative correlation between EP300-AS1 and overall survival (OS) of LUAD (**e**) and LUSC (**f**) patients in TCGA dataset. **g**, **h** EP300-AS1 expression was detected in LUAD tissues (**g**) or LUSC tissues (**h**) and their adjacent normal tissues using the specific EP300-AS1 FISH probe. Histograms displayed quantification of EP300-AS1 fluorescence intensity using a fluorescence microscope, with 9 different fields of vision evaluation by Image J software analysis. Scale bar, 50 μm. **P < 0.01. **i** The association between EP300-AS1 expression with OS or progression-free survival (FPS) of lung cancer patients using Kaplan-Meier Plotter website (https://kmplot.com/analysis/). **j**, **k** The relationship between EP300-AS1 expression and clinicopathological features in LUAD (**j**) and LUSC (**k**) patients in TCGA dataset. *P < 0.05, **P < 0.01.

To identify metastasis-related lncRNAs, gene expression profiles of human LUAD CL1-0 and its highly metastatic subpopulation CL1-5 cells from GSE42407 dataset were analyzed. In this dataset, 51 upregulated and 66 downregulated lncRNAs were identified in highly metastatic CL1-5 cells (Fig. 1a). Since LUAD and LUSC account for the majority of NSCLC cases, TCGA-LUAD and TCGA-LUSC datasets were used to explore common molecular changes in NSCLC. In TCGA-LUAD dataset, compared with normal tissues, 327 upregulated and 158 downregulated lncRNAs were identified in LUAD tissues (Fig. 1b). In TCGA-LUSC dataset, 432 upregulated and 215 downregulated lncRNAs were identified in LUSC tissues (Fig. 1c). Among these differentially expressed lncRNAs, MIR210HG and LINC01116 were considered as commonly upregulated lncRNAs, and EP300-AS1 and SFTA1P were considered as commonly downregulated lncRNAs (Fig. 1d). Univariate Cox regression analysis found that EP300-AS1 expression was negatively associated with overall survival (OS) with significance in both LUAD and LUSC (Fig. 1e, f). To validate EP300-AS1 expression in LUAD and LUSC tissues, 32 LUAD and 26 LUSC tissues and matched normal tissues were collected for FISH analysis. Consistent with the bioinformatics results, EP300-AS1 expression was lower in LUAD and LUSC tissues than in normal tissues (Fig. 1g, h).

In addition, high EP300-AS1 expression was significantly associated with longer overall survival (OS) and progression-free survival (FPS) in patients with lung cancer using Kaplan-Meier Plotter website (Fig. 1i). And High EP300-AS1 expression was significantly associated with longer OS and disease-free survival (DFS) in TCGA-LUAD patients (Fig. S1a). However, a trend of association between high EP300-AS1 expression with longer OS and DFS was observed in TCGA-LUSC patients, despite the lack of statistical significance (Fig. S1b). To determine whether EP300-AS1 is an independent prognostic factor for NSCLC patients, we conducted a multivariate survival analysis. This analysis integrated EP300-AS1 and other clinicopathological features, including tumor size (T), lymph node metastasis (N), pathologic stage, age, and gender. However, owing to the insufficient data on distant metastasis (M), this variable could not be incorporated into the multivariate analysis. For LUAD patients, tumor size, pathologic stage and EP300-AS1 exerted significant effects on prognosis (Fig. S1c). Only EP300-AS1 expression served as a significantly independent factor for prognosis prediction for LUSC patients (Fig. S1d). Although lymph node metastasis (N stage) and pathological stage were included, the absence of M stage data also led to an incomplete multi-factor survival analysis, forcing us to collect clinicopathological data in the future. Compared to those with early stage, EP300-AS1 expression was significantly reduced in LUAD patients with advanced clinical stage (Fig. 1j). EP300-AS1 expression was also decreased in LUAD patients with distant metastasis. Similar patterns were observed in LUSC patients from TCGA dataset (Fig. 1k). These results suggest that EP300-AS1 is a vital lncRNA associated with NSCLC metastasis and progression.

### EP300-AS1 suppresses NSCLC cell proliferation, migration and invasion

**Fig. 2:**
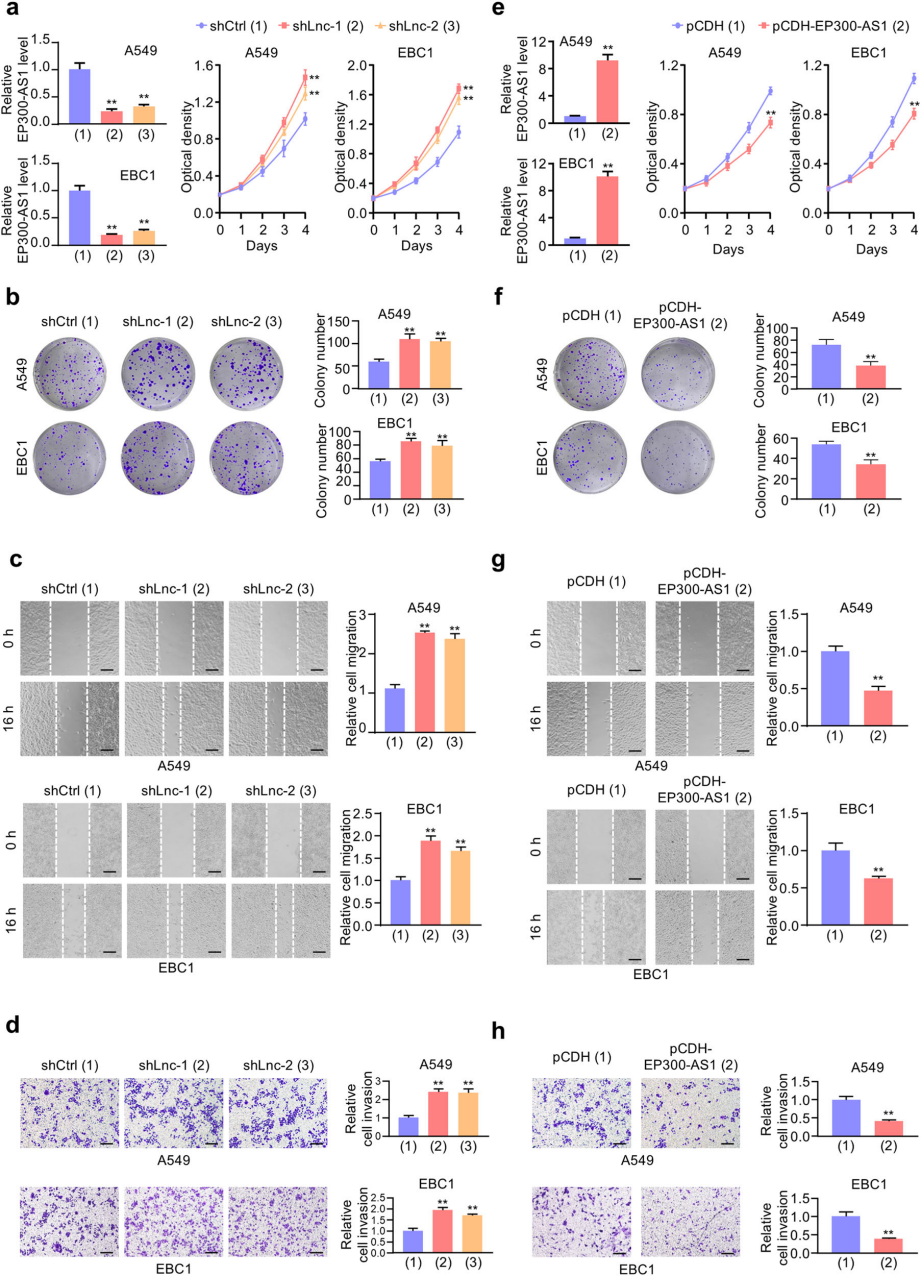
EP300-AS1 suppresses NSCLC cell proliferation, migration and invasion. **a** A549 and EBC1 cells were stably infected with recombinant lentiviruses carrying control shRNA (shCtrl) or two independent shRNAs targeting EP300-AS1 (shLnc-1 or shLnc-2), and cell numbers was detected by CCK8 assay at specified times. Relative EP300-AS1 expression was determined by qRT-PCR (*n* = 3). **P < 0.01. **b** Colony formation assays for A549 and EBC1 cells infected as in (**a**) (*n* = 3). **P < 0.01. **c**, **d** Wound-healing assays (**c**) and transwell assays (**d**) for A549 and EBC1 cells infected as in (**a**) (*n* = 3). Scale bar, 100 μm. **P < 0.01. **e**, **f** A549 and EBC1 cells were stably infected with empty pCDH vector or pCDH-EP300-AS1 and analyzed as in (**a,** **b**) (*n* = 3). **P < 0.01. **g**, **h** Wound-healing assays (**g**) and transwell assays (**h**) for A549 and EBC1 cells infected as in (**e**) (*n* = 3). Scale bar, 100 μm. **P < 0.01. Data shown are mean ± SD.

To investigate the biological role of EP300-AS1 in NSCLC cells, LUAD A549 and LUSC EBC1 cells were infected with recombinant lentiviruses carrying control shRNA (shCtrl) or two independent shRNAs targeting EP300-AS1 (shLnc-1 or shLnc-2). Cell proliferation and colony formation assays revealed that A549 and EBC1 cells with shLnc-1 or shLnc-2 grew faster compared to control cells (Fig. 2a, b). Wound-healing and transwell assays showed that EP300-AS1 knockdown enhanced migration and invasion of A549 and EBC1 cells (Fig. 2c, d). Conversely, compared to those with empty pCDH vector, the proliferation, migration, and invasion abilities of A549 and EBC1 cells stably infected with pCDH-EP300-AS1 were reduced (Fig. 2e, f, g, h). These results suggest that EP300-AS1 has ability to inhibit NSCLC cell proliferation, migration and invasion in vitro.

Next, adenoviruses carrying EP300-AS1 (Ad.EP300-AS1) were generated using pADM-CMV-C-3Flag-mCMV-copGFP vector (Fig. S2a). NSCLC cells were infected with Ad.EP300-AS1 at 0, 10, 50, 100 and 500 multiplicity of infection (MOI). The results showed that Ad.EP300-AS1 increased EP300-AS1 expression and inhibited A549 and EBC1 cell proliferation in a dose-dependent manner (Fig. S2b, c). To further evaluate the anti-tumor effect of Ad.EP300-AS1 in vivo, NTG mice were subcutaneously injected with approximately 1 × 10^6^ A549 cells. After twelve days, PBS or Ad.EP300-AS1 (5 × 10^8^ pfu or 1 × 10^9^ pfu) was injected intratumorally for a total of five doses. The results showed that Ad.EP300-AS1 inhibited A549 tumor growth in vivo (Fig. S2d). These findings suggest that EP300-AS1 has therapeutic potential for NSCLC.

### EP300-AS1 directly interacts with RNA-binding protein PTBP1

**Fig. 3:**
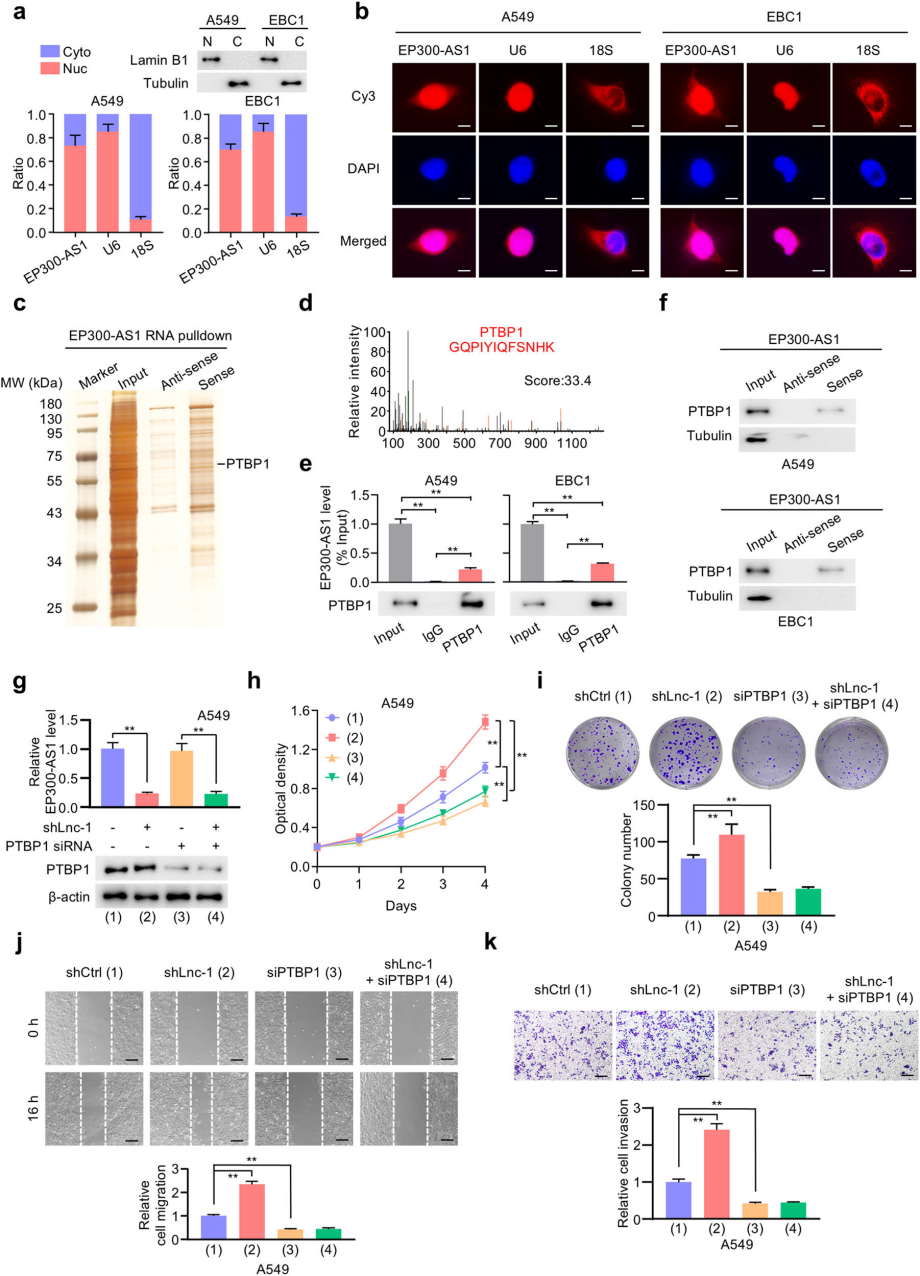
EP300-AS1 directly interacts with PTBP1 protein. **a** The ratio of nuclear and cytoplasmic EP300-AS1 expression in A549 and EBC1 cells was detected by qRT-PCR (*n* = 3). Lamin B1 as the nucleus marker and tubulin as the cytoplasm marker were estimated. **b** Subcellular localization of EP300-AS1 was detected using FISH. Scale bar, 10 μm. **c** Cellular extracts from A549 cells were precipitated by the sense and antisense of EP300-AS1 using extracellular F2-RNA pulldown assay, followed by SDS-PAGE and silver staining. The differential protein bands were analyzed by mass spectrometry (MS). MW, molecular weight. **d** MS profile of EP300-AS1-binding protein PTBP1 with score. **e** RIP assays were performed using antibodies against PTBP1 in A549 and EBC1 cells (*n* = 3). **P < 0.01. **f** Intracellular F2-RNA pulldown assays were performed using the sense and antisense of EP300-AS1, followed by immunoblot (IB) (*n* = 3). **P < 0.01. **g** A549 cells stably expressing shCtrl or EP300-AS1 shRNA (shLnc-1) were transfected with control siRNA or PTBP1 siRNA as indicated. PTBP1 expression were examined by IB, and EP300-AS1 expression were examined by qRT-PCR (*n* = 3). **P < 0.01. **h**, **i** CCK8 assays (**h**) and colony formation assays (**i**) for A549 cells treated as in (**g**) (*n* = 3). **P < 0.01. **j**, **k** Wound-healing assays (**j**) and transwell assays (**k**) for A549 cells infected as in (**g**) (*n* = 3). Scale bar, 100 μm. **P < 0.01. Data shown are mean ± SD.

Subcellular fractionation assays showed that EP300-AS1 was mainly localized in the nucleus of A549 and EBC1 cells, which was further confirmed by FISH assays (Fig. 3a, b). To identify EP300-AS1-binding proteins, extracellular RNA pulldown assays combined with mass spectrometry were performed. Silver staining revealed specific differentially expressed bands (Fig. 3c). Polypyrimidine tract binding protein 1 (PTBP1), a nuclear RNA-binding protein, was identified as an EP300-AS1-binding partner with high score (Fig. 3d). RIP assays further demonstrated that EP300-AS1 was significantly enriched in PTBP1 immunoprecipitates in A549 and EBC1 cells (Fig. 3e). Intracellular RNA pulldown assays also confirmed the interaction between PTBP1 and EP300-AS1 in A549 and EBC1 cells (Fig. 3f). Thus, we transfected shCtrl and shLnc-1 cells with control siRNA or PTBP1 siRNA as indicated. Western blot analysis showed that EP300-AS1 knockdown did not affect PTBP1 protein levels, and qRT-PCR indicated that silencing PTBP1 could not influence EP300-AS1 expression in A549 and EBC1 cells (Fig. 3g and Fig. S3a). Interestingly, cell proliferation and colony formation assays showed that silencing PTBP1 inhibited and abolished EP300-AS1 knockdown-promoted A549 and EBC1 cell proliferation (Fig. 3h, i and Fig. S3b, c). Furthermore, wound-healing and transwell assays demonstrated that silencing PTBP1 also abrogated EP300-AS1 knockdown-promoted migration and invasion of A549 and EBC1 cells (Fig. 3j, k and Fig. S3d, e). We transfected shCtrl and shLnc-1 cells with empty vector or FLAG-PTBP1. Consistent with the results of PTBP1 silencing, PTBP1 overexpression promoted A549 and EBC1 cell proliferation without altering EP300-AS1 expression (Fig. S4a–d). Wound-healing and transwell assays confirmed that PTBP1 overexpression promoted A549 and EBC1 cell migration and invasion (Fig. S4e–h). Notably, EP300-AS1 knockdown did not increase proliferation, migration, or invasion in PTBP1-overexpressing NSCLC cells. These results collectively indicate that PTBP1 is functionally involved in EP300-AS1-mediated NSCLC development.

### EP300-AS1-PTBP1 axis regulates PRMT5 mRNA stability

**Fig. 4:**
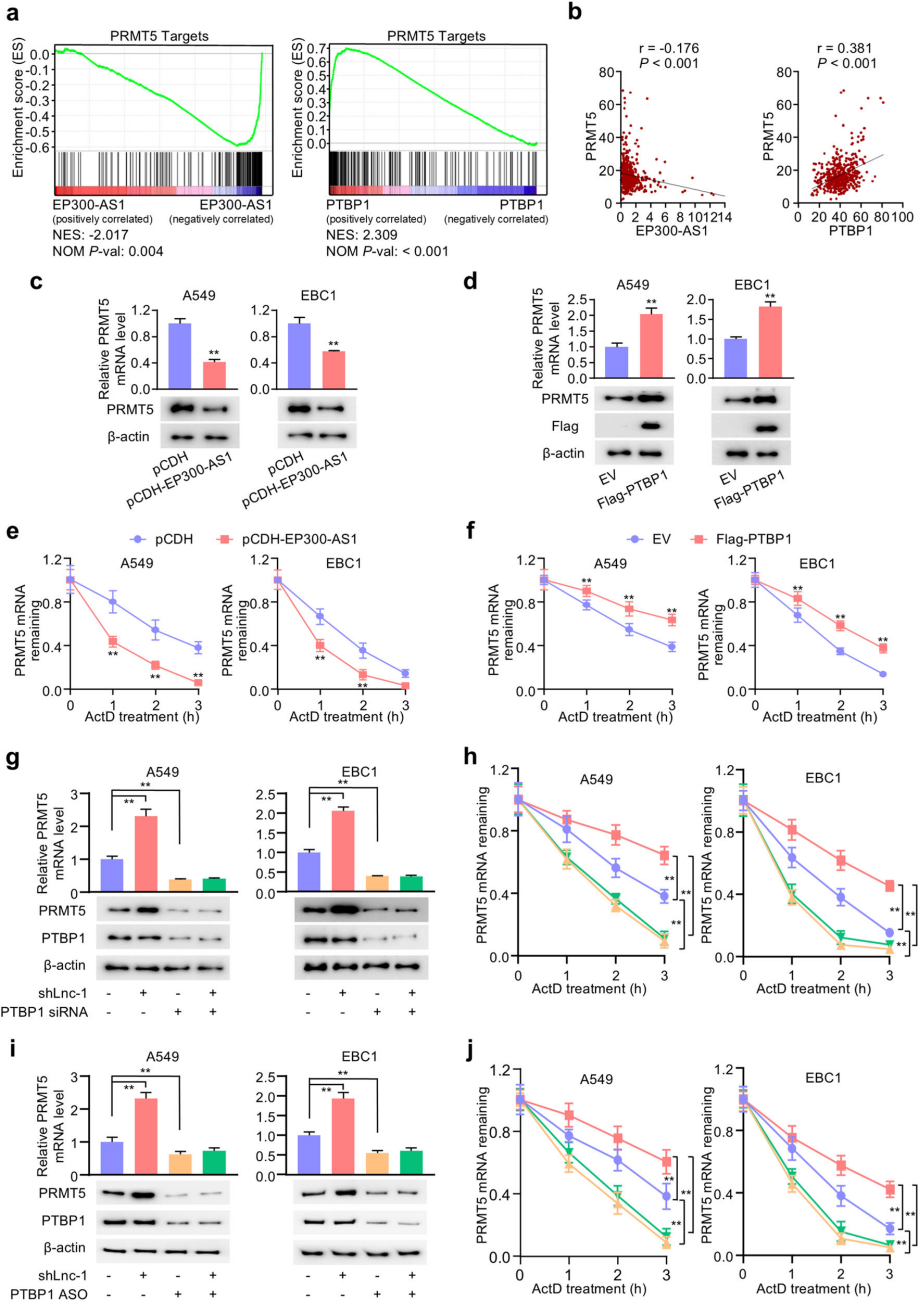
EP300-AS1 interacts with PTBP1 to downregulate PRMT5 mRNA stability. **a** GSEA was conducted to predict the function of EP300-AS1 or PTBP1 based on TCGA-LUAD dataset. **b** The correlation between EP300-AS1 or PTBP1 expression and PRMT5 mRNA expression based on TCGA-LUAD dataset. **c** Relative PRMT5 mRNA level in A549 and EBC1 cells stably expressing pCDH and pCDH-EP300-AS1 were examined by qRT-PCR (*n* = 3). **P < 0.01. **d** Relative PRMT5 mRNA level in A549 and EBC1 cells transfected with empty vector (EV) and FLAG-PTBP1 were examined by qRT-PCR (*n* = 3). **P < 0.01. **e** PRMT5 mRNA in A549 and EBC1 cells stably expressing pCDH and pCDH-EP300-AS1 were examined by qRT-PCR at the indicated times after 10 µg/ml actinomycin D (ActD) (*n* = 3). **P < 0.01. **f** PRMT5 mRNA in A549 and EBC1 cells transfected with EV and FLAG-PTBP1 were examined by qRT-PCR at the indicated times after 10 µg/ml ActD (n = 3). **P < 0.01. **g** A549 and EBC1 cells stably expressing shCtrl or shLnc-1 were transfected with control siRNA or PTBP1 siRNA as indicated. IB and qRT-PCR analysis examined PRMT5 protein and mRNA expression (*n* = 3). **P < 0.01. **h** PRMT5 mRNA in A549 and EBC1 cells treated as in (**g**) were examined by qRT-PCR at the indicated times after 10 µg/ml ActD (*n* = 3). **P < 0.01. **i** A549 and EBC1 cells stably expressing shCtrl or shLnc-1 were transfected with control ASO or PTBP1 ASO as indicated. IB and qRT-PCR analysis examined PRMT5 protein and mRNA expression (n = 3). **P < 0.01. **j** PRMT5 mRNA in A549 and EBC1 cells treated as in (**i**) were examined by qRT-PCR at the indicated times after 10 µg/ml ActD (*n* = 3). **P < 0.01. Data shown are mean ± SD.

To explore the mechanisms through which EP300-AS1 and PTBP1 function in NSCLC, Gene Set Enrichment Analysis (GSEA) was performed. The results showed that EP300-AS1 expression was negatively correlated with PRMT5 target genes, while PTBP1 mRNA level was positively correlated (Fig. 4a). Additionally, EP300-AS1 expression was negatively correlated with PRMT5 mRNA expression, whereas PTBP1 mRNA was positively correlated with PRMT5 mRNA based on TCGA dataset (Fig. 4b). Given that PRMT5 is widely recognized as a crucial oncogene in various malignant cancers, including NSCLC [29–31], we investigated whether the EP300-AS1-PTBP1 axis could regulate PRMT5 expression in NSCLC cells. Compared to control groups, EP300-AS1 overexpression reduced PRMT5 mRNA and protein levels, while PTBP1 overexpression increased those level both in A549 and EBC1 cells (Fig. 4c, d). Importantly, RNA decay assays further demonstrated that PRMT5 mRNA stability was significantly decreased in EP300-AS1-overexpressing cells and increased in PTBP1-overexpressing cells (Fig. 4e, f). Conversely, EP300-AS1 knockdown enhanced PRMT5 mRNA stability and protein level, while silencing PTBP1 inhibited and abolished these effects in A549 and EBC1 cells (Fig. 4g, h). Additionally, we have transfected A549 and EBC1 cells with PTBP1-targeting ASO to silencing PTBP1 expression. PTBP1 ASO also inhibited and abrogated EP300-AS1 knockdown-enhanced PRMT5 mRNA stability and level (Fig. 4i, j).

MRTX1719 selectively inhibits PRMT5 and PRMT5-promoted tumor activity, and is currently undergoing phase I/II clinical trials [32]. Next, shCtrl and shLnc-1 cells were treated with DMSO or MRTX1719. Compared with control group, MRTX1719 inhibited cell proliferation without affecting EP300-AS1 expression. Importantly, MRTX1719 abolished EP300-AS1 knockdown-promoted A549 and EBC1 cell proliferation (Fig. S5a–d). Wound-healing and transwell assays showed that MRTX1719 treatment also inhibited and reversed the enhanced migration and invasion caused by EP300-AS1 knockdown in both cell lines (Fig. S5e–h). In addition, shCtrl or shLnc-1 NSCLC cells were co-transfected with PTBP1 siRNA and empty vector or FLAG-PRMT5. PTBP1 silencing inhibited and abolished the effects of EP300-AS1 on PRMT5 expression and cellular behavior. However, PRMT5 overexpression promoted NSCLC cell proliferation, migration and invasion without altering EP300-AS1 or PTBP1 expression. Neither EP300-AS1 nor PTBP1 affected proliferation, migration, or invasion in PRMT5-overexpressing NSCLC cells (Fig. S6a–d). These results suggest that the EP300-AS1-PTBP1 axis regulates NSCLC progression through modulation of PRMT5 mRNA stability and expression.

### EP300-AS1 inhibits PTBP1 cytoplasmic translocation and PRMT5 mRNA-PTBP1 complex formation

**Fig. 5:**
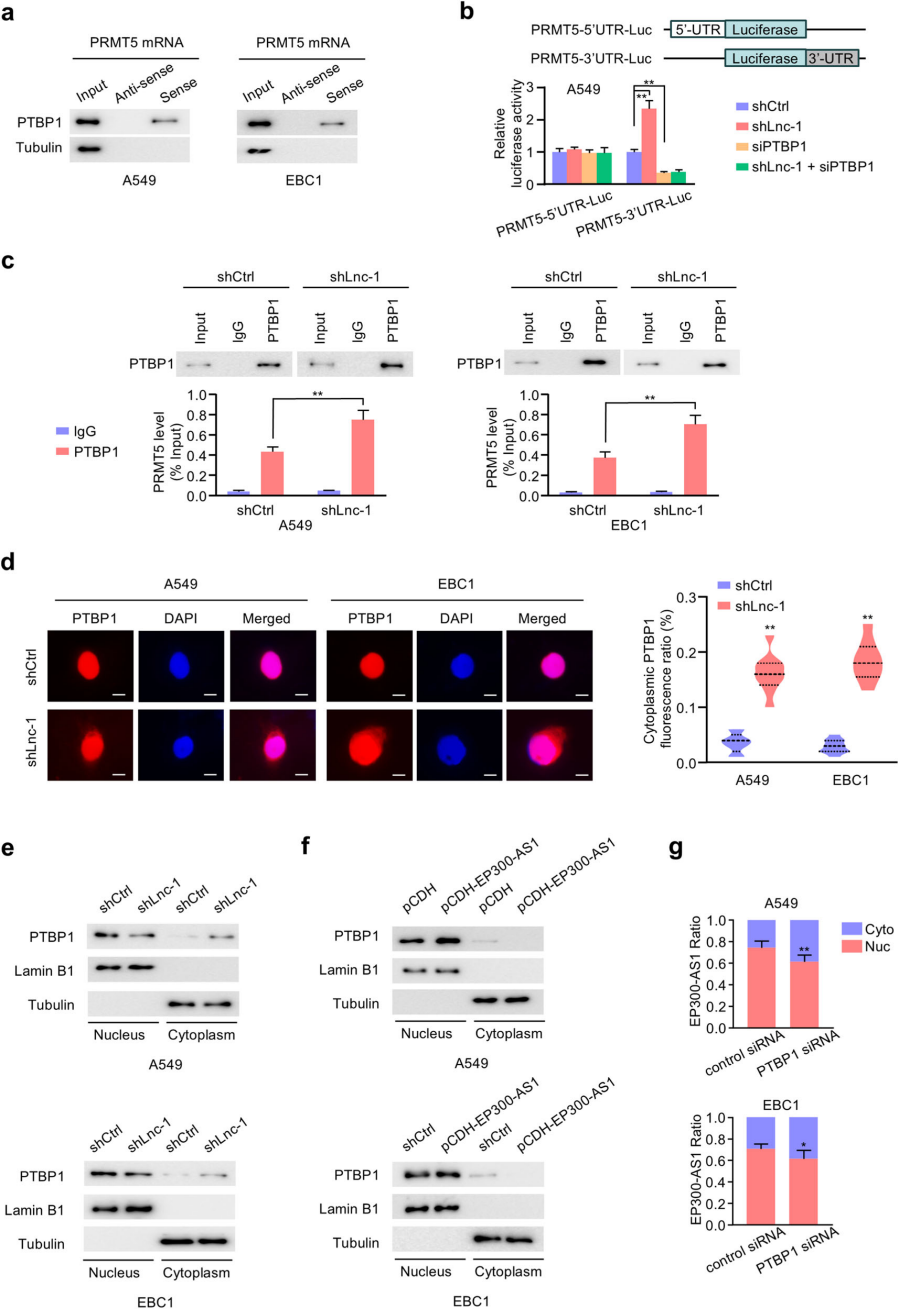
Silencing EP300-AS1 promotes PTBP1 cytoplasmic translocation and PRMT5 mRNA-PTBP1 interaction. **a** Extracellular F2-RNA pulldown assay were performed using the sense and antisense of PRMT5 mRNA, followed by IB (*n* = 3). **b** Schematic diagram of the luciferase reporter plasmids with 5’-UTR or 3’-UTR sequences of PRMT5. Luciferase reporter assays were performed in A549 cells stably expressing shCtrl or shLnc-1 and transfected with control siRNA or PTBP1 siRNA and indicated luciferase reporter plasmids (*n* = 3). **P < 0.01. **c** RIP assays were performed using antibodies against PTBP1 in A549 and EBC1 cells stably expressing shCtrl or shLnc-1 (*n* = 3). **P < 0.01. **d** Immunofluorescence analysis of subcellular localization of PTBP1 in A549 and EBC1 cells stably expressing shCtrl or shLnc-1. Scale bar, 10 μm. Histograms displayed quantification of cytoplasmic PTBP1 fluorescence intensity using a fluorescence microscope, with 9 different fields of vision evaluation by Image J software analysis. **P < 0.01. **e** IB assay of nuclear and cytoplasmic PTBP1 protein in A549 and EBC1 cells stably expressing shCtrl or shLnc-1 (*n* = 3). **f** IB assay of nuclear and cytoplasmic PTBP1 protein in A549 and EBC1 cells stably expressing pCDH or pCDH-EP300-AS1 (*n* = 3). **g** The ratio of nuclear and cytoplasmic EP300-AS1 expression in A549 and EBC1 cells transfected with control siRNA or PTBP1 siRNA was detected by qRT-PCR (*n* = 3). *P < 0.05, **P < 0.01. Data shown are mean ± SD.

PTBP1, a member of the polypyrimidine tract-binding protein family, shuttles between cell nucleus and cytoplasm [33]. In the cytoplasm, PTBP1 regulates mRNA stability by binding to 3’-UTR [34]. RNA pulldown assays showed that PTBP1 interacted with PRMT5 mRNA in A549 and EBC1 cells (Fig. 5a). To explore the degradation pathway of PRMT5 mRNA, A549 and EBC1 cells were transfected with control siRNA, RRP6 siRNA or XRN1 siRNA. RRP6, as a core subunit of exosome complex, plays an important role in 3’-5’ mRNA degradation pathway. And XRN1 is involved in 5’-3’ mRNA degradation. Compared with control group, both XRN1 knockdown and RRP6 knockdown increased PRMT5 mRNA levels, and RRP6 knockdown had a more pronounced effect. Importantly, PTBP1 silencing did not affect PRMT5 mRNA level in cells with RRP6 knockdown, suggesting that PTBP1 regulates PRMT5 mRNA stability mainly through 3’-5’ degradation pathway (Fig. S7a). To confirm whether PTBP1-enhanced PRMT5 mRNA stability depends on 3’-UTR region, luciferase reporter plasmids containing PRMT5 mRNA 5’-UTR or 3’-UTR were generated. We co-transfected shCtrl and shLnc-1 cells with control siRNA or PTBP1 siRNA and the luciferase reporters. Luciferase assays revealed that EP300-AS1 knockdown increased luciferase activity in cells with PRMT5-3’UTR-Luc, while silencing PTBP1 reduced and abolished this activity. No changes were observed in cells with PRMT5-5’UTR-Luc plasmids (Fig. 5b). These results revealed that PTBP1 interacted with PRMT5 mRNA 3’-UTR and prevented its 3’-5’ degradation.

RIP assays were then used to assess the effect of EP300-AS1 on the PTBP1-PRMT5 mRNA interaction. In shCtrl cells, PRMT5 mRNA was significantly enriched in PTBP1 immunoprecipitates compared to IgG. This enrichment was further increased in EP300-AS1-silenced cells (Fig. 5c). Conversely, EP300-AS1 overexpression disrupted the PTBP1-PRMT5 mRNA complex in A549 and EBC1 cells (Fig. S7b). Since both EP300-AS1 and PTBP1 are predominantly localized in cell nucleus, we investigated whether EP300-AS1 affected the subcellular localization of PTBP1. Immunofluorescence analysis showed that EP300-AS1 knockdown increased cytoplasmic PTBP1 fluorescence ratios, which was further verified by subcellular fractionation assays in A549 and EBC1 cells (Fig. 5d, e). Conversely, EP300-AS1 overexpression decreased PTBP1 cytoplasmic localization in both cell lines (Fig. 5f). However, in PTBP1-silenced cells, cytoplasmic PTBP1 levels were minimal and not increased by EP300-AS1 knockdown (Fig. S7c). PTBP1 knockdown also increased cytoplasmic localization of EP300-AS1 in A549 and EBC1 cells (Fig. 5g). These results suggest that EP300-AS1 not only prevents PTBP1 cytoplasmic translocation, but also blocks PTBP1-PRMT5 mRNA interaction.

We next investigated the precise molecular mechanisms by which EP300-AS1 prevents PTBP1 cytoplasmic translocation. As reported, PTBP1 protein posttranslational modification, including acetylation and phosphorylation, could modulate its nucleocytoplasmic transport [35, 36]. Therefore, we explored whether EP300-AS1 had ability to modulate PTBP1 acetylation or phosphorylation. The results showed that EP300-AS1 knockdown or overexpression did not affect PTBP1 acetylation (Fig. S7d, e). However, EP300-AS1 knockdown increased PTBP1 phosphorylation, whereas EP300-AS1 overexpression decreased PTBP1 phosphorylation in NSCLC cells (Fig. S7f, g). The phosphorylation and nuclear-cytoplasmic transport of PTBP1 protein were directly regulated by cAMP-dependent protein kinase (PKA) [36]. To assess whether EP300-AS1 influences PTBP1-PKA interactions, shCtrl and shLnc-1 cells were transfected with FLAG-PTBP1 and MYC-tagged PKA catalytic subunit alpha (PKA C-alpha). Co-immunoprecipitation confirmed the interaction between PTBP1 and PKA C-alpha in A549 and EBC1 cells. EP300-AS1 knockdown enhanced this interaction, while EP300-AS1 overexpression weakened it (Fig. S7h, i). These findings suggest that EP300-AS1 prevents PTBP1 cytoplasmic translocation through inhibiting PKA-mediated PTBP1 phosphorylation.

### Silencing EP300-AS1 promotes A549 tumor growth and metastasis in vivo

**Fig. 6:**
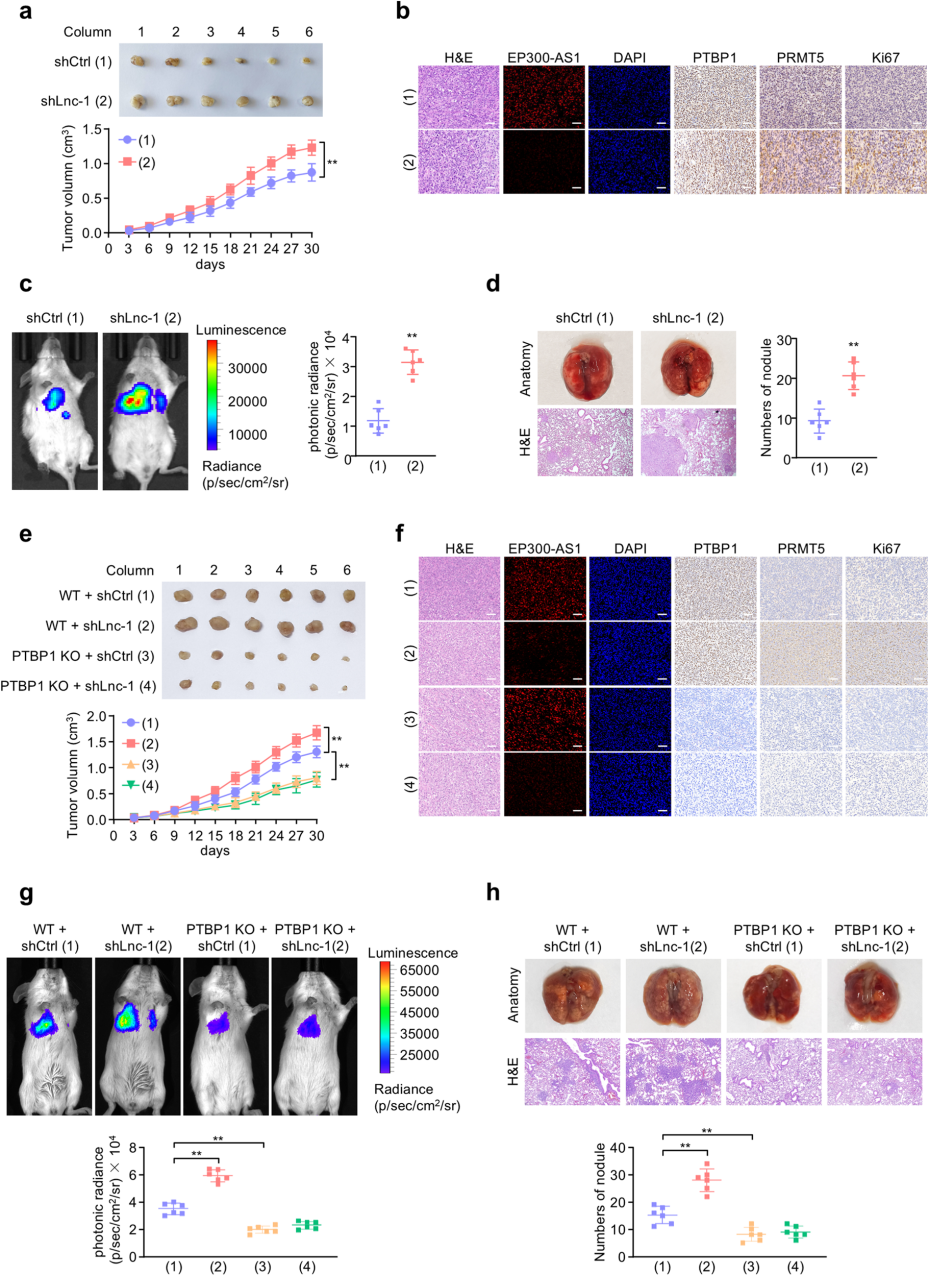
Silencing EP300-AS1 promotes A549 tumor growth and metastasis in NTG mice. **a** A549 cells stably expressing shCtrl or shLnc-1 were injected subcutaneously in the right flank of NTG mice, and tumor volume was measured with vernier-caliper at the indicated times (*n* = 6). **P < 0.01. **b** Representative FISH of EP300-AS1 and immunohistochemical (IHC) staining of PRMT5, PTBP1 and Ki67 expression in A549 tumor tissues. Scale bar, 50 μm. **c** Representative bioluminescence image of NTG mice injected with A549 cells stably expressing shCtrl or shLnc-1 and firefly luciferase by tail vein at 30 days. The luminescence signal intensities were represented by overlaid false-color images and quantitatively analyzed (*n* = 6). **P < 0.01. **d** Representative lung tissues and H&E-stained tissue sections from (**c**). The number of tumor nodules are shown. **P < 0.01. **e** WT or PTBP1 KO EBC1 cells stably expressing shCtrl or shLnc-1 were injected subcutaneously in the right flank of NTG mice, and tumor volume was measured as in (**a**) (*n* = 6). **P < 0.01. **f** Representative FISH of EP300-AS1 and immunohistochemical (IHC) staining of PRMT5, PTBP1 and Ki67 expression in EBC1 tumor tissues. Scale bar, 50 μm. **g** Luminescence signal intensities of NTG mice injected with WT or PTBP1 KO EBC1 cells stably expressing shCtrl or shLnc-1 and firefly luciferase by tail vein were quantitatively analyzed as in (**c**) (*n* = 6). **P < 0.01. **h** Representative lung tissues and H&E-stained tissue sections from (**g**). **P < 0.01. Data shown are mean ± SD.

To investigate the role of EP300-AS1 in tumor growth in vivo, A549 cells stably expressing EP300-AS1 shRNA or control shRNA were subcutaneously injected into the right flank of NTG mice. Compared with control group, EP300-AS1-silenced A549 cells formed significantly larger tumors (Fig. 6a). Immunohistochemical analysis revealed that EP300-AS1 knockdown increased PRMT5 and Ki67-positive cell number without affecting PTBP1 levels, supporting the tumor-suppressive role of EP300-AS1 (Fig. 6b). To evaluate the effect of EP300-AS1 on metastasis in vivo. NTG mice injected with A549 cells stably expressing EP300-AS1 shRNA or control shRNA and firefly luciferase via tail vein. Bioluminescence imaging showed significantly higher luminescence signals in the lung regions of the EP300-AS1 knockdown group (Fig. 6c). H&E staining further confirmed an increased number of tumor nodules in the lungs of these mice (Fig. 6d).

In addition, shCtrl or shLnc-1 EBC1 cells were used to generate PTBP1 knockout (KO) cells via CRISPR/Cas9. Wild-type (WT) or PTBP1 KO EBC1 cells stably expressing shCtrl or shEP300-AS1 were subcutaneously injected into the right flank of NTG mice. EP300-AS1 knockdown promoted EBC1 tumor growth, while PTBP1 KO inhibited and abolished this effect (Fig. 6e). Immunohistochemical staining showed that EP300-AS1 knockdown increased PRMT5 and Ki67-positive cell number without changing PTBP1 expression. PTBP1 KO reduced and eliminated the increase in PRMT5- and Ki67-positive cells induced by EP300-AS1 knockdown (Fig. 6f). Additionally, NTG mice were injected with WT or PTBP1 KO EBC1 cells stably expressing shCtrl or shEP300-AS1 and firefly luciferase via tail vein. Luminescence signals in the lung region were significantly increased in the EP300-AS1 shRNA group, while PTBP1 KO inhibited and abolished EP300-AS1 knockdown-induced tumor metastasis (Fig. 6g). H&E staining of the lungs also confirmed these findings (Fig. 6h).

### Correlation between EP300-AS1 and PRMT5 expression in human NSCLC patients

**Fig. 7:**
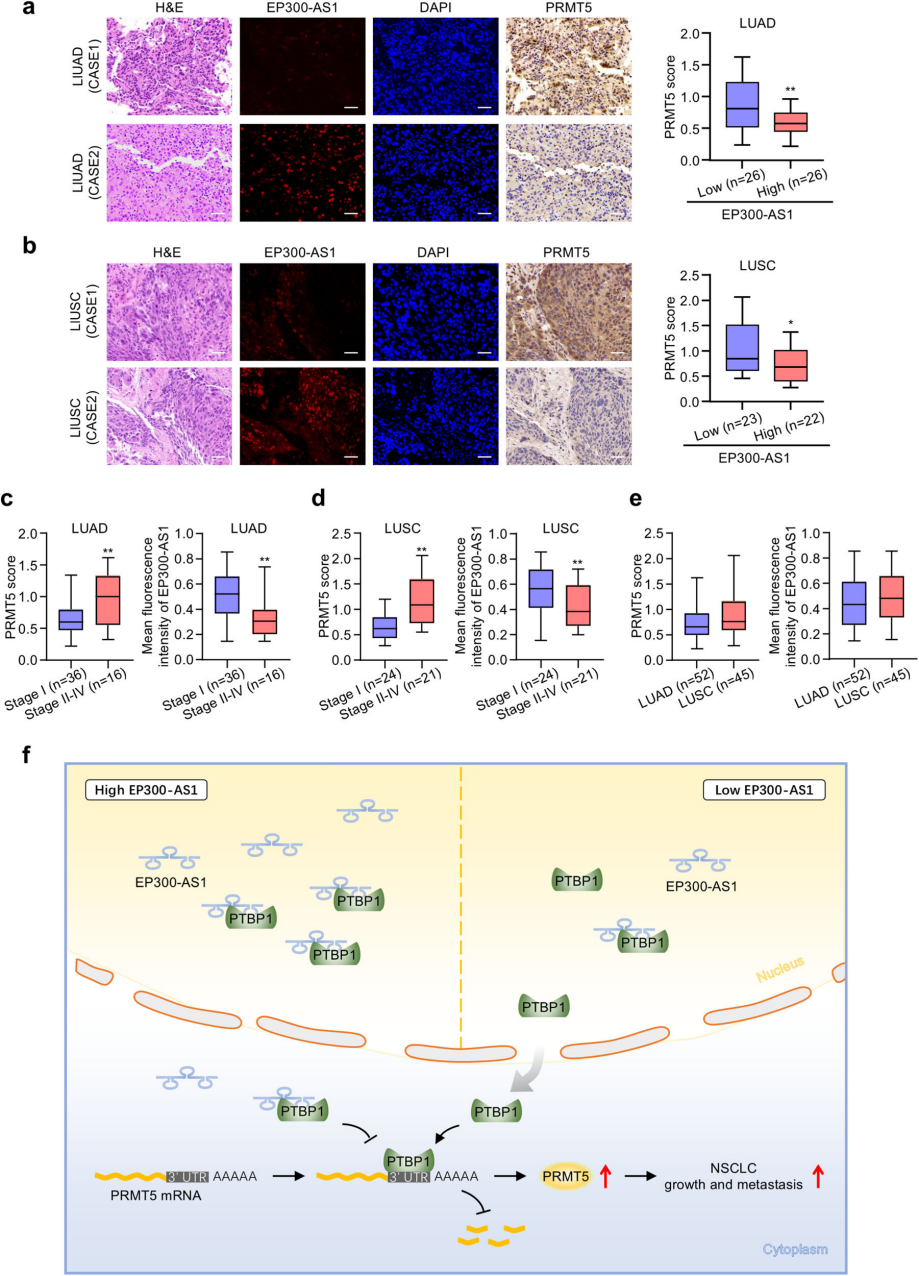
Correlation between EP300-AS1 and PRMT5 expression in human NSCLC patients. **a**, **b** Representative FISH of EP300-AS1 and IHC of PRMT5 expression in human LUAD tissues (**a**) or LUSC tissues (**b**). Histograms displayed PRMT5 expression in the low or high EP300-AS1 expression groups. Scale bar, 50 μm. *P < 0.05, **P < 0.01. **c**, **d** The relationship between clinical stage and EP300-AS1 or PRMT5 expression in LUAD (**c**) and LUSC (**d**) patients. **P < 0.01. **e** The relationship between NSCLC pathological subtypes and EP300-AS1 or PRMT5 expression. **f** A proposed model depicted that EP300-AS1 prevented PTBP1 cytoplasmic translocation and PRMT5 mRNA-PTBP1 complex formation, thus damaged PRMT5 mRNA stability and inhibited NSCLC growth and metastasis.

We further collected 52 human LUAD cases and 45 human LUSC cases. The characteristics of 97 NSCLC patients were listed in Table S1. EP300-AS1 expression was assessed by FISH, and PRMT5 expression was evaluated by immunohistochemistry. Consistent with TCGA database analysis and experimental results in vitro, EP300-AS1 expression was negatively correlated with PRMT5 expression in both LUAD and LUSC tissues (Fig. 7a, b). The relationship between clinical stage or pathological subtype and the expression of EP300-AS1 and PRMT5 was further analyzed. Compared to early-stage tumors, EP300-AS1 expression was significantly reduced and PRMT5 expression was significantly increased in advanced-stage LUAD and LUSC tissues, respectively (Fig. 7c, d). No significant differences in EP300-AS1 or PRMT5 expression were observed between LUAD and LUSC (Fig. 7e).

We further analyzed EP300-AS1 expression in multiple human cancers from GSE149507 and TCGA datasets. Compared to the corresponding normal tissues, EP300-AS1 expression was significantly decreased in SCLC tissues, whereas no significant differences in EP300-AS1 expression were observed in colon adenocarcinoma (COAD), stomach adenocarcinoma (STAD), liver hepatocellular carcinoma (LIHC), breast carcinoma (BRCA), or kidney renal clear cell carcinoma (KIRC) (Fig. S8a). These data suggest that EP300-AS1 expression exhibits tumor type-specific characteristics. Additionally, we further transfected SCLC-21H cells with empty vector or EP300-AS1. Compared to control groups, EP300-AS1 overexpression reduced PRMT5 mRNA and protein levels without altering PTBP1 expression (Fig. S8b). And EP300-AS1 overexpression inhibited SCLC-21H cell proliferation and invasion (Fig. S8c, d). EP300-AS1 also interacted with PTBP1, and prevented its cytoplasmic translocation and PTBP1-PRMT5 mRNA complex formation in SCLC-21H cells (Fig. S8e–g). However, due to the lack of clinical data in SCLC patients, the role of EP300-AS1 in SCLC progression needs to be further investigated. The specificity of EP300-AS1 probe was confirmed by FISH, the specificity of PRMT5 antibody was confirmed by immunohistochemistry, and the specificity of PTBP1 antibody was confirmed by immunoblotting using cell lysates (Fig. S9). Taken together, these data demonstrate a significant pathological correlation between EP300-AS1 and PRMT5 in NSCLC.

## Discussion

Significant progress has been made in understanding the biology and mechanisms of NSCLC progression, contributing to improvements in diagnostic accuracy and treatment strategies. However, the prognosis for NSCLC patients remains poor, particularly in advanced stages or metastatic disease [37]. Therefore, identifying new molecular targets and mechanisms is essential to improving clinical outcomes. This study identified the EP300-AS1-PTBP1-PRMT5 axis as a critical regulator of NSCLC growth and metastasis, highlighting the clinical relevance of EP300-AS1 in NSCLC (Fig. 7f). EP300-AS1 expression was lower in NSCLC tissues compared to normal tissues and was associated with advanced clinical stage, distant metastasis, and poor prognosis. EP300-AS1 suppressed NSCLC cell proliferation, migration, invasion and metastasis both in cultured cells and NTG mice. Mechanistically, EP300-AS1 prevented PTBP1 cytoplasmic translocation and disrupted PTBP1-PRMT5 mRNA complex formation, thereby reducing PRMT5 mRNA stability and expression. Overall, this study established the physiological and pathological significance of the EP300-AS1-PTBP1-PRMT5 axis in NSCLC progression, suggesting that EP300-AS1 mimetics or PTBP1 inhibition may represent promising therapeutic strategies for NSCLC.

LncRNA refers to a type of RNA transcript longer than 200 nucleotides that does not encode proteins. Most lncRNAs maintain their stability by being capped and polyadenylated [38, 39]. A lncRNA locus can regulate chromatin structure or gene transcription in *cis* through several mechanisms, including recruiting transcriptional regulatory factors, exerting gene regulatory effects or containing DNA regulatory elements [5, 40–42]. In trans, lncRNAs perform diverse biological functions by binding to proteins, DNA, or RNA molecules [43–45]. These interactions influence gene expression, cellular processes and cancer development by modifying molecular behavior. Yang et al. reported that LINC02159 interacted with Aly/REF export factor (ALYREF), enhancing YAP1 mRNA stability through 5-methylcytosine modification [46]. Elevated YAP1 levels activated the Hippo and β-catenin signaling pathways, contributing to NSCLC progression. In another study, YY1-induced lncRNA PKMYT1AR promoted expression of the oncogene PKMYT1 by sponging miR-485-5p in NSCLC cells [47]. However, the entire network and mechanisms of lncRNAs regulating NSCLC progression remain largely unknown. Although EP300-AS1 has been briefly mentioned in bioinformatics studies as potentially related to LUAD prognosis, this study comprehensively investigated its biological functions and molecular mechanisms in NSCLC for the first time. EP300-AS1 reduced the mRNA stability and expression of the oncogene PRMT5 by interacting with PTBP1 protein, thereby inhibiting NSCLC growth and metastasis.

PTBP1 is a widely recognized RNA-binding protein that regulates gene expression through various post-transcriptional mechanisms, including mRNA splicing, translation, stability, and localization [48]. Under different physiological and pathological conditions, PTBP1 can shuttle between the nucleus and cytoplasm. However, no study has reported a small molecule that directly inhibits PTBP1 activity. The multikinase inhibitor PT109 was found to suppress glioblastoma proliferation by indirectly reducing PTBP1 expression. In this study, PTBP1 knockout inhibited EBC1 tumor growth in vivo, providing a foundation for developing PTBP1 inhibitors for NSCLC treatment [49]. Recently, several lncRNAs have been reported to function by binding to PTBP1 [50–52]. Miao et al. showed that MALAT1 regulated pre-mRNA alternative splicing by stabilizing the interaction between PTBP1 and PTB-associated splicing factor (PSF) in hepatocellular carcinoma [53]. LncRNA FIRRE was identified to interact with PTBP1 by RNA pulldown and RIP assays. The interaction induced PTBP1 cytoplasmic translocation and stabilized BECN1 mRNA in a PTBP1-dependent manner, thereby promoting colorectal cancer development [54]. LncRNA SNHG5 stabilized autophagy related 5 (ATG5) mRNA and induced cancer-associated fibroblasts-like phenotype by interaction with PTBP1 in acute myeloid leukemia cells [55]. And lncRNA AC006064.4-201 bound to PTBP1 and blocked PTBP1-CDKN1B mRNA interaction, thereby destabilizing CDKN1B mRNA and alleviating senescence in chondrocytes [56]. Our study hypothesized and confirmed that EP300-AS1 is a novel PTBP1-binding RNA. Additionally, PRMT5 mRNA was identified as a novel PTBP1-binding target in this study. PTBP1 stabilized PRMT5 mRNA through binding to its 3’-UTR. EP300-AS1 prevented PTBP1 cytoplasmic translocation and blocked PTBP1-PRMT5 mRNA complex formation. PRMT5, as a well-established oncogene, plays a crucial role in NSCLC development [57]. We further found that the regulatory function of the EP300-AS1-PTBP1 axis depended on PRMT5 mRNA stability and expression in NSCLC. Although this study comprehensively demonstrated the existence of the EP300-AS1-PTBP1-PRMT5 axis in NSCLC, whether EP300-AS1 binds to other proteins requires further investigation.

This study demonstrated that EP300-AS1 acted as a promising target involved in NSCLC progression. EP300-AS1 expression was decreased in NSCLC compared to normal tissues, and negatively associated with OS and DFS. Therefore, FISH could be further used to test EP300-AS1 expression to predict clinical outcomes in NSCLC patients. Next, adenoviruses carrying EP300-AS1 inhibited NSCLC growth in a dose-dependent manner both in vitro and in vivo, suggesting that EP300-AS1 had therapeutic potential for NSCLC. And small molecules for enhancing EP300-AS1-PTBP1 interaction or synthetic EP300-AS1 mimics might also become relevant from a therapeutic perspective in the future. However, our study primarily screened out EP300-AS1 as a crucial regulatory lncRNA of NSCLC growth and metastasis based on TCGA and GEO datasets. These databases, although providing invaluable data for our research endeavors, inherently entail specific limitations, such as regional differences in sample sources and inconsistencies in data processing methods. This regional variability implies that the bioinformatic findings might not be generalizable to other populations with distinct sample characteristics, limiting the scope of conclusions. Inconsistencies in data processing methods may also lead to discrepancies in the comparability and reliability of bioinformatic data. To address these limitations, this study examined the biological function and molecular mechanism of EP300-AS1 during NSCLC progression using functional experiments. Additionally, our study collected 97 human NSCLC cases to examine the associations of EP300-AS1 with the clinicopathological features of NSCLC patients. Therefore, EP300-AS1 may serve as a promising potential biomarker and therapeutic target for NSCLC diagnosis and treatment.

In conclusion, this study comprehensively demonstrates the anti-tumor biological function and molecular mechanism of EP300-AS1 in NSCLC. EP300-AS1 inhibits NSCLC cell proliferation, invasion and metastasis both in vitro and in vivo by reducing PRMT5 mRNA stability in a PTBP1-dependent manner. EP300-AS1 expression is low and negatively correlated with PRMT5 expression in NSCLC tissues. These findings outline the EP300-AS1-PTBP1-PRMT5 as a key regulatory axis in NSCLC progression.

## Materials and methods

### Cell lines, plasmids, lentivirus, adenovirus, RNA oligonucleotides, and regents

Human LUAD A549 cells and LUSC EBC1 cells were purchased from the American Type Culture Collection (ATCC) and tested for mycoplasma contamination. Cells were routinely cultured in DMEM (Invitrogen, USA) containing 25 mM glucose and 10% fetal bovine serum (FBS) at 37 °C. When cells reached approximately 70% confluence, they were transfected with plasmids or siRNAs using Lipofectamine 3000 reagent (Invitrogen, USA) or Lipofectamine RNAiMAX (Invitrogen, USA), respectively. Luciferase reporter plasmids were constructed by inserting PRMT5 5’-UTR or 3’-UTR into the pGL3 vector (Promega, USA). The cDNA target sequences of siRNAs and/or shRNAs for EP300-AS1 and PTBP1 were listed in Table S2. Lentiviral vectors for gene overexpression were obtained by inserting PCR-amplified sequences into the pCDH vector (System Biosciences, USA). Lentiviral shRNA vectors were constructed by cloning shRNA fragments into the pSIH-H1-Puro vector (System Biosciences, USA). Adenoviruses carrying EP300-AS1 (Ad.EP300-AS1), constructed using the pADM-CMV-C-3Flag-mCMV-copGFP vector, were purchased from Vigene Biosciences. PTBP1 knockout cells were generated using the CRISPR/Cas9 system. The single guide RNA sequence targeting human PTBP1 was CCTCTAGAGTGATCCACATC. The PRMT5 inhibitor MRTX1719 (2630904-45-7) was purchased from Topscience. Anti-PTBP1 (12582-1-AP), anti-PRMT5 (18436-1-AP), anti-XRN1 (23108-1-AP), anti-RRP6 (11178-1-AP), and anti-Lamin B1 (12987-1-AP) antibodies were purchased from Proteintech, anti-β-actin (sc-47778HRP) and anti-MYC (sc-40HRP) was purchased from Santa Cruz, and anti-Flag (A8592) was purchased from Sigma-Aldrich.

### Data collection and analysis

RNA-seq datasets from 515 TCGA-LUAD tissues and 59 normal tissues, and 501 TCGA-LUSC tissues and 49 normal tissues, were obtained from The Cancer Genome Atlas (TCGA, www.cancergenome.nih.gov). Gene expression profiles of human LUAD CL1-0 cells and highly metastatic CL1-5 were obtained from GSE42407 dataset [58]. Differentially expressed lncRNAs in GSE42407, TCGA-LUAD and TCGA-LUSC datasets were analyzed using the limma R package, with significance thresholds of false discovery rate (FDR) < 0.05 and |log2 fold change (FC)| > 1. Univariate Cox regression analysis was conducted to identify prognosis-related lncRNAs. Survival analysis of EP300-AS1 in lung cancer patients was performed using Kaplan-Meier Plotter website (https://kmplot.com/analysis/), and survival analysis in TCGA-LUAD and TCGA-LUSC patients with follow-up within 5 years was conducted using the survival R package. For multi-factor survival analysis, EP300-AS1 and the other clinicopathological features, including tumor size (T), lymph node metastasis (N), pathologic stage, age, and gender, were integrated. Although there are 505 cases of TCGA-LUSC patients, only 7 patients have definite metastatic data (M1 stage) and 84 patients lack M stage data. Similarly, among 523 cases of TCGA-LUAD patients, only 25 patients have definite metastatic data (M1 stage) and 145 patients lack M stage data. In order to ensure the accuracy of other clinicopathological factors, the insufficient M stage data objectively fails to meet the statistical requirement for the number of events in the multi-factor survival analysis. For clinicopathological analysis, tissues classified as N0 and M0 were defined as non-metastatic, while those with N1-3 or M1 stage were defined as having metastatic predisposition.

### Cell proliferation, migration and invasion assays

Cell proliferation was measured by the CCK8 reagent (Dojindo) according to the manufacturer’s instruction. For colony formation assays, cells were seeded in a 6-well plate at a density of 2 × 10^3^ cells per well. After 10 days, cells were fixed and stained with 0.1% crystal violet solution. The number of colonies larger than 1.5 mm in diameter was counted. Cell migration ability was assessed by wound-healing assays. After the cell density reached approximately 80%, a pipette tip was used to create a scratch. Floating cells were washed away with PBS. After 16 h culture, photos were taken again at the initial location to calculate the distance of cell migration. Cell invasion was detected by transwell assays. Briefly, 200 μL cell suspension with serum-free was added into the upper chamber coated with matrigel. After 16 h culture, the cells were fixed and stained with 0.1% crystal violet solution. The number of invaded cells was quantified using Image J software.

### Luciferase reporter assay

Cells were seeded in 24-well plates and transfected with luciferase reporter plasmids and the indicated siRNAs using Lipofectamine 3000 or Lipofectamine RNAiMAX. After 48 hours, cells were analyzed for luciferase and β-galactosidase activities according to the manufacturer’s instruction (Promega, USA). Briefly, cells were harvested and resuspended in lysis buffer to ensure complete lysis. The lysate was transferred to a tube and centrifuged at 1.2 × 10^4 ^r/min for 5 min at 4 °C. The supernatant was mixed with an equal volume of luciferase assay reagent, and luciferase activity was measured using a luminometer. For β-galactosidase activity assay, the supernatant was transferred to a 96-well plate and incubated with the assay buffer for 1 h at 4 °C. The absorbance was measured at 420 nm using a microplate reader.

### RNA isolation and quantitative real-time PCR (qRT-PCR)

Total RNA was extracted using TRIzol reagent (Invitrogen, USA) and reverse-transcribed into cDNA using the Quantscript RT Kit (Tiangen, China), following the manufacturer’s protocol. Quantitative real-time PCR was conducted using SYBR Premix Ex Taq (Takara, Japan) to measure gene expression. Relative expression levels were normalized to α-Tubulin and calculated using the comparative Ct method. Primer sequences used for qRT-PCR were listed in Table S3.

### RNA decay assay

Cells were seeded into six-well plates at a density of 5 × 10^5^ cells per well and allowed to adhere overnight. Then, cells were treated with DMSO or 10 µg/mL mRNA transcription inhibitor actinomycin D. RNA was isolated at the indicated time points using TRIzol reagent (Invitrogen, USA), following the manufacturer’s protocol. Quantitative real-time PCR was conducted using SYBR Premix Ex Taq (Takara, Japan) to the stability of PRMT5 mRNA.

### Subcellular fractionation

Nuclear and cytoplasmic RNA or proteins were extracted using the PARIS™ Kit (Invitrogen, USA) according to the manufacturer’s instructions. Briefly, cells were collected and resuspended in cell fractionation buffer on ice for 10 min. The samples were centrifuged at 3000 r/min for 2 min at 4 °C to separate nuclear and cytoplasmic fractions. The supernatant containing the cytoplasmic fraction was collected into a fresh RNase-free tube, and the nuclear pellet was lysed with cell disruption buffer on ice. The resulting lysates were used for protein analysis, and RNA was extracted using lysis/binding solution.

### Fluorescence in situ hybridization assay

Cells were seeded onto slides and incubated at 37 °C overnight. Slides were rinsed once with PBS and fixed with 4% paraformaldehyde for 10 minutes. After permeabilization and pre-hybridization, the slides were hybridizated with FISH probes in a humidified chamber at 37 °C in the dark overnight. Slides were washed three times with wash buffer and counterstained with DAPI in the dark for 10 min. Human EP300-AS1, U6, and 18S probes were designed and synthesized by RiboBio (China). For paraffin-embedded tissue sections, lncRNA FISH was performed using the Ribo FISH Kit (RiboBio, China) following the manufacturer’s instructions. All images were captured using a fluorescence microscope (Nikon, Japan).

### RNA pull-down analysis

The F2 tag is a short RNA sequence containing seventeen nucleotides (GGCGCTGACAAAGCGC). It could fuse with target RNA sequence, having minimal impact on target RNA structure or function. For extracellular RNA pulldown assay, PCR was performed to obtain T7-sense F2-labeled RNA and T7-antisense F2-labeled RNA transcription templates, using the target DNA sequence as a template. Based on the F2-labeled RNA transcription templates, F2-labeled RNA was synthesized using the T7 High Yield RNA Transcription Kit (Vazyme, China). The reaction was incubated at 37 °C for 4 h to yield the sense and antisense F2-labeled RNA. Then, the reaction complex was incubated with 1 μL DNase I at 37 °C for 15 min to digest DNA template. The 3 μg F2-labeled RNA was incubated with cell lysates and performed for RNA pull-down assays using the RNA pulldown Kit (Fitgene, China) following the manufacturer’s protocol. For intracellular F2-RNA pulldown assay, the eukaryotic expression plasmids encoding F2-labeled RNA were constructed based on the pcDNA3.0 vector (Invitrogen, USA). A549 or EBC1 cells were transfected with these plasmids and cultured for 24 hours. Cells were then lysed and processed using the RNA pulldown Kit (Fitgene, China). Precipitated proteins were separated by SDS-PAGE, visualized with silver staining, and analyzed by mass spectrometry (MS).

### RNA immunoprecipitation (RIP) assay

RIP was performed using the Magna RIP™ RNA-Binding Protein Immunoprecipitation Kit (Millipore, USA) following the manufacturer’s protocol to identify protein-bound RNAs. Briefly, cells were collected and resuspended in an equal pellet volume of complete RIP lysis buffer on ice. Remove 10 µL cell supernatant for “Input”. Magnetic beads were completely dispersed by pipetting and transferred to each tube with 50 µL per tube. The magnetic beads were washed twice with 500 µL RIP wash buffer, and were incubated with anti-PTBP1 antibody or control IgG on a rotating platform for 30 min. Then, the cell supernatant was immunoprecipitated using the magnetic bead-antibody complex on a rotating platform overnight at 4 °C. The magnetic beads were washed six times with 500 µL RIP wash buffer, and mixed with salt solution for one hour at −80 °C to precipitate RNA. The immunoprecipitated RNA was analyzed by qRT-PCR.

### Analysis of tumor growth and metastasis in vivo

All animal experiments were approved by the Ethical Committee of Shengjing Hospital of China Medical University. No blinding was performed in this study. In all experiments, the mice were fed the normal diet (Sibeifu Biotechnology, China) and housed in an environment with a 12 h light/dark cycle, a constant temperature of 21–23 °C, and a relative humidity of 50–60%. Six-week-old female NTG mice (Sibeifu Biotechnology, China) were randomly divided into different groups. Approximately 1 × 10^6^ A549 cells stably expressing shCtrl or EP300-AS1 shRNA (shLnc-1) were subcutaneously injected into the right flank of 6 NTG mice in each group. Mice were euthanized at the specified time. Tumor volumes were measured using a vernier caliper at the indicated time points, and tumor growth curve was charted. The resected tumors were used for FISH and immunohistochemical (IHC) staining analysis. For tumor metastasis assay, each NTG mice injected with approximately 2 × 10^6^ A549 cells stably expressing shCtrl or shLnc-1 and firefly luciferase by tail vein. Mice were imaged at day 30 using the IVIS200 Imaging System (Xenogen Corporation, USA), and luminescence signals in the lungs were quantitatively analyzed. Lung tissues were collected and examined histologically after euthanasia.

### Human clinical samples

Fifty-two primary LUAD and adjacent normal tissues, and forty-five LUSC and adjacent normal tissues, were collected from Shengjing Hospital of China Medical University with informed consent of patients and with the approval of the Ethical Committee of Shengjing Hospital of China Medical University. Fresh tissues were used for immunohistochemical staining with anti-PRMT5 and anti-Ki67 antibodies. PRMT5 and Ki67 staining scores were analyzed using the IHC Profiler plugin in ImageJ software.

### Statistical analysis

Trial experiments or similar experiments done previously were used to assess sample size with adequate statistical power. For comparisons between two groups, Student’s t-test was used for parametric data, and the Mann-Whitney U test was used for nonparametric data. One-way ANOVA with Bonferroni correction was applied for multiple group comparisons. Overall survival and disease-free survival curves were generated using the Kaplan-Meier method, and differences between survival curves were assessed with the log-rank test. All statistical tests were two-sided. Analyses were conducted using SPSS 13.0 and GraphPad Prism 9.0 software. The *P*-value < 0.05 was considered statistically significant.

## Supplementary information

Supplementary Figure1-8

Supplementary Table1-3

Original data files

## Data Availability

Experimental data and full immunoblots supporting the conclusions of this study are available within the Article and the Supplementary Material. All databases used in this study are publicly available.
