# Supplementary material for: LncRNA EP300-AS1 interacts with PTBP1 to destabilize PRMT5 mRNA and suppresses NSCLC growth and metastasis
Source: Cell Death Dis. 2025 Aug 11;16(1):607. doi: 10.1038/s41419-025-07931-3 (PMC12339964; doi:10.1038/s41419-025-07931-3)
Supplement: Supplementary file 1 — Supplementary Figure1-8 [file 41419_2025_7931_MOESM1_ESM.pdf]

Figure S1

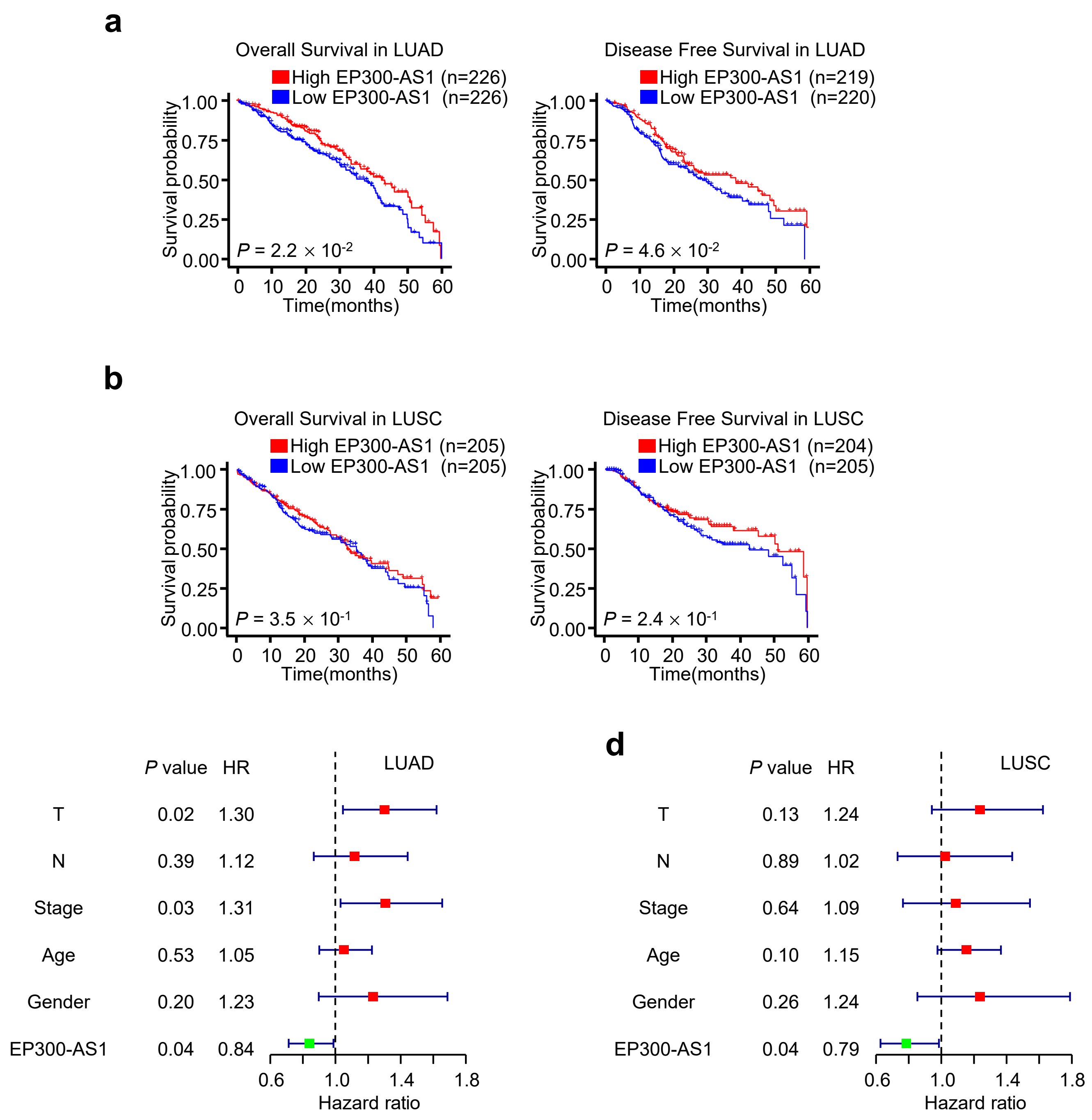

Supplementary Fig.1 The association between EP300-AS1 and clinical outcome in NSCLC patients

a The over survival (OS) and disease-free survival (DFS) curve related to EP300-AS1 in LUAD patients from TCGA-LUAD dataset. **b** The OS and DFS curve related to EP300-AS1 in LUSC patients from TCGA-LUSC dataset. **c, d** Multivariate factor survival analysis of EP300-AS1 and other other clinicopathological factors of LUAD (**c**) and LUSC (**d**) patients in TCGA dataset.

Figure S2

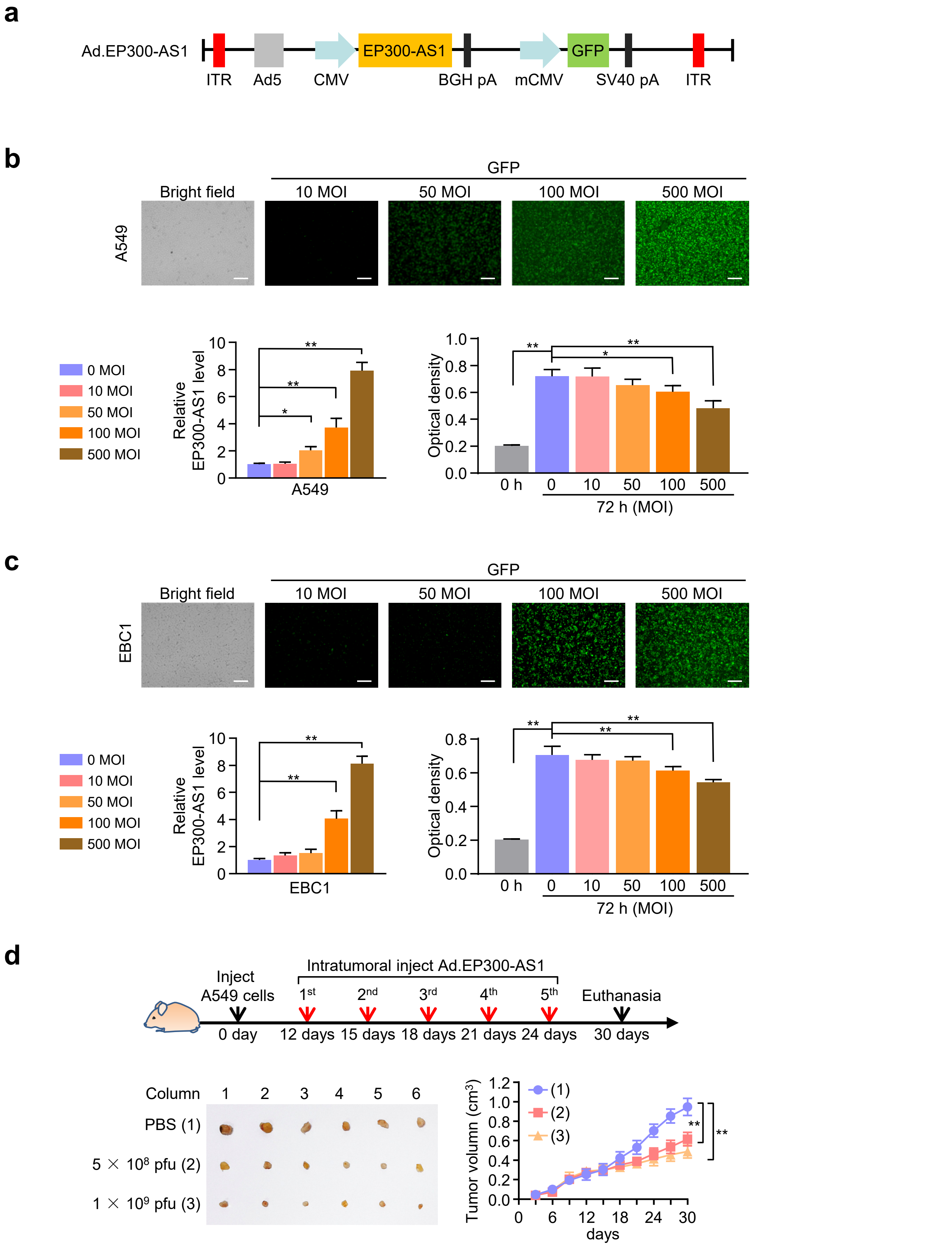

Supplementary Fig.2 Adenoviruses harboring EP300-AS1 inbihit NSCLC cell proliferation

a Diagram of the recombinant adenovirus construct expressing EP300-AS1 (Ad.EP300-AS1). PA, poly(A) signal. ITR, inverted terminal repeat. **b, c** CCK8 assays for A549 (**b**) and EBC1 (**c**) cells infected with Ad.EP300-AS1. Infection efficiencies were monitored by fluorescence. EP300-AS1 expression was examined by qRT-PCR (n=3). Scale bar, 100 μm. *P < 0.05, **P < 0.01. **d** A549 cells were injected subcutaneously in the right flank of NTG mice. After twelve days, PBS or Ad.EP300-AS1 were injected intratumorally for a total of five doses as indicated. and tumor volume was measured with vernier-caliper at the indicated times (n=6). **P < 0.01. Data shown are mean ± SD.

Figure S3

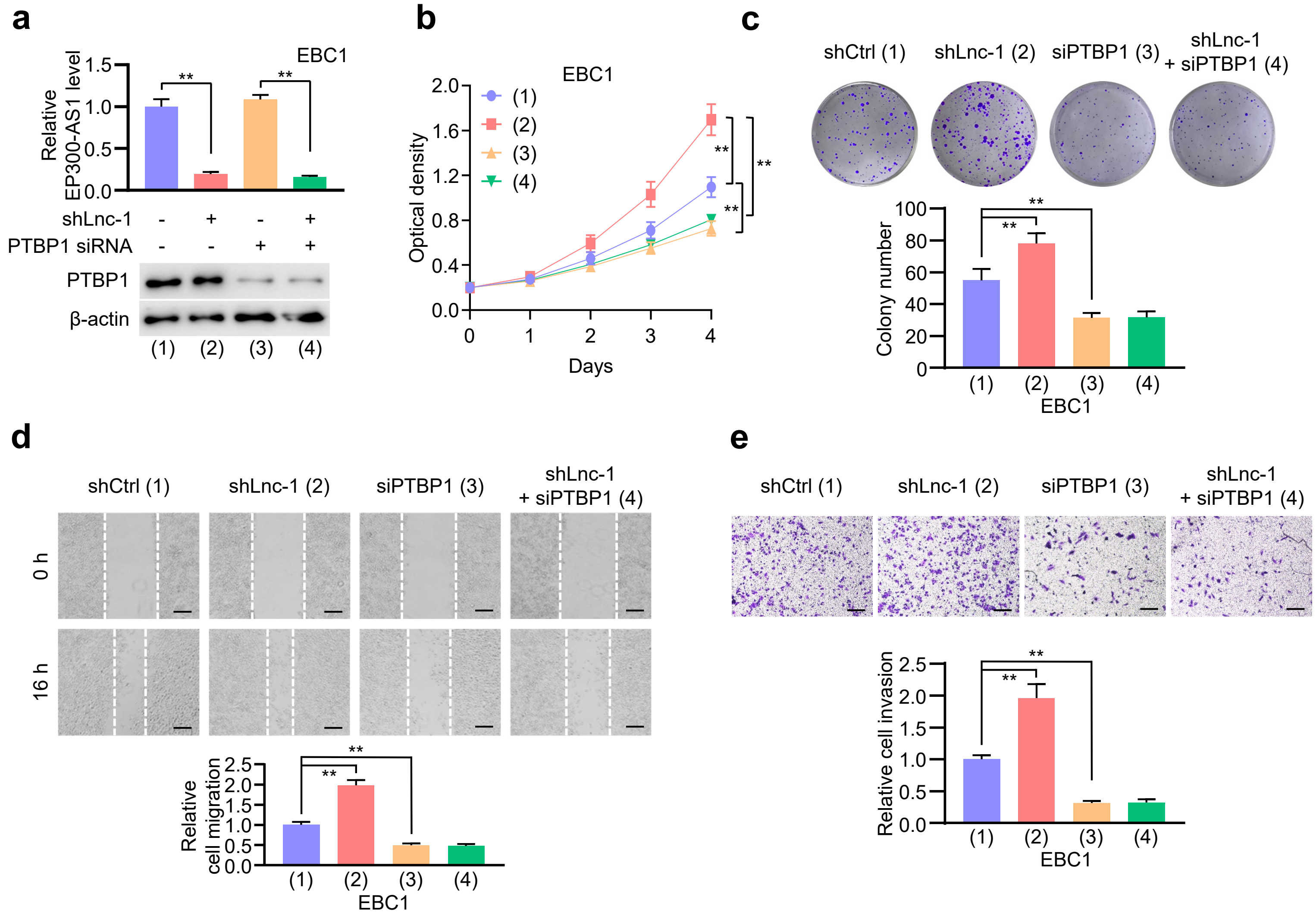

Supplementary Fig.3 EP300-AS1 inbihit EBC1 cell proliferation, migration and invasion via PTBP1

a EBC1 cells stably expressing shCtrl or EP300-AS1 shRNA (shLnc-1) were transfected with control siRNA or PTBP1 siRNA as indicated. PTBP1 expression were examined by IB, and EP300-AS1 expression were examined by qRT-PCR (n=3). $**P < 0.01$. **b, c** CCK8 assays (**b**) and colony formation assays (**c**) for EBC1 cells treated as in (**a**) (n=3). $**P < 0.01$. **d, e** Wound-healing assays (**d**) and transwell assays (**e**) for EBC1 cells treated as in (**a**) (n=3). Scale bar, 100 μm. $**P < 0.01$. Data shown are mean ± SD.

Figure S4

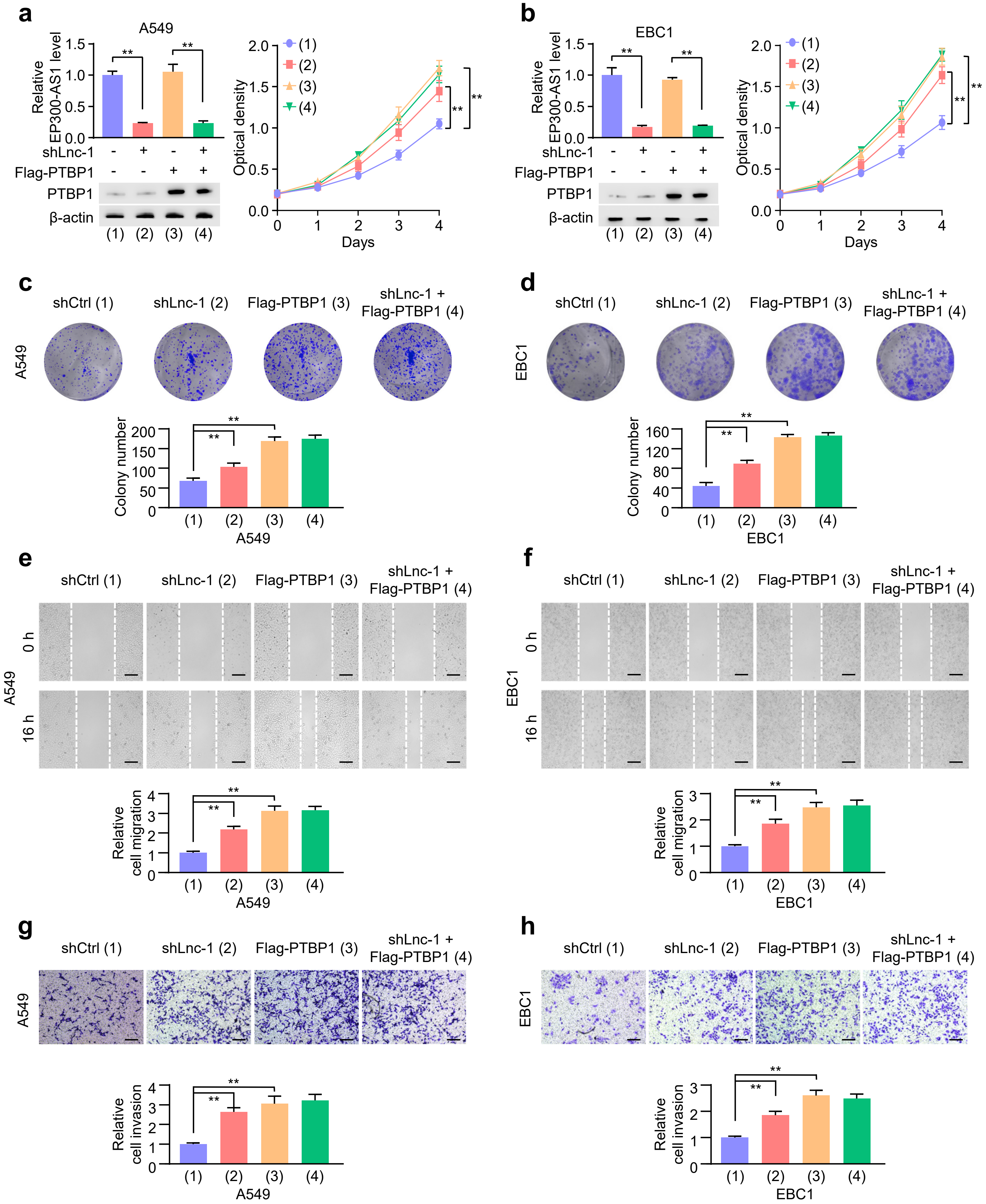

Supplementary Fig.4 PTBP1 promotes NSCLC cell proliferation, migration and invasion

a, b CCK8 assays for A549 (**a**) and EBC1 (**b**) cells stably expressing shCtrl or shLnc-1 and transfected with empty vector or FLAG-PTBP1. EP300-AS1 expression was examined by qRT-PCR (n=3). ***P* < 0.01. **c, d** Colony formation assays for A549 (**c**) and EBC1 (**d**) cells treated as in (**a**) (n=3). ***P* < 0.01. **e, f** Wound-healing assays for A549 (**e**) and EBC1 (**f**) cells treated as in (**a**) (n=3). Scale bar, 100 μm. ***P* < 0.01. **g, h** Transwell assays for A549 (**g**) and EBC1 (**h**) cells treated as in (**a**) (n=3). Scale bar, 100 μm. ***P* < 0.01. Data shown are mean ± SD.

Figure S5

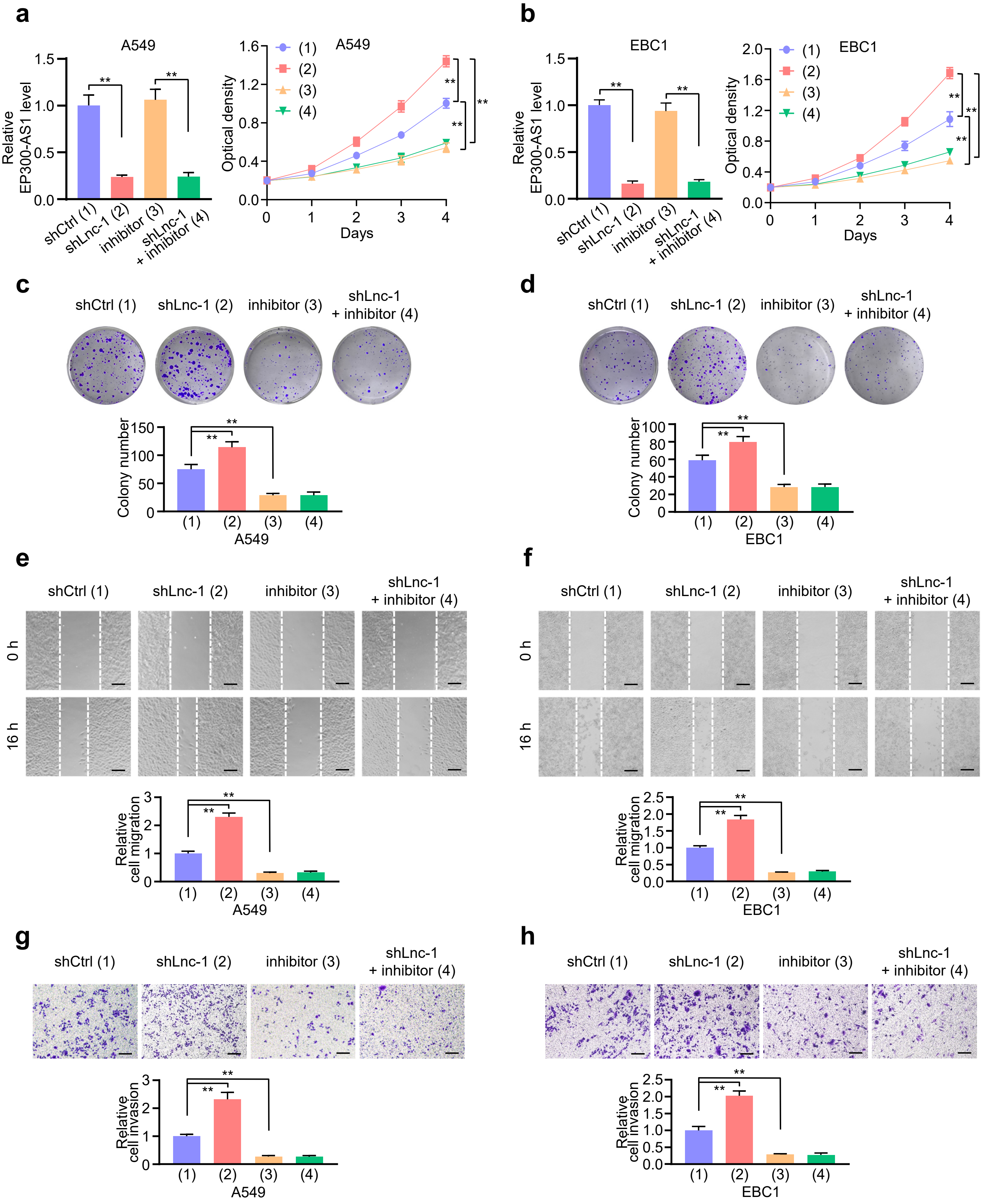

Supplementary Fig.5 PRMT5 inhibitor MRTX1719 inbihits NSCLC cell proliferation, migration and invasion

a, b CCK8 assays for A549 (**a**) and EBC1 (**b**) cells stably expressing shCtrl or shLnc-1 and treated with 10 nM PRMT5 inhibitor MRTX1719. EP300-AS1 expression was examined by qRT-PCR (n=3). ***P* < 0.01. **c, d** Colony formation assays for A549 (**c**) and EBC1 (**d**) cells treated as in (**a**) (n=3). ***P* < 0.01. **e, f** Wound-healing assays for A549 (**e**) and EBC1 (**f**) cells treated as in (**a**) (n=3). Scale bar, 100 μm. ***P* < 0.01. **g, h** Transwell assays for A549 (**g**) and EBC1 (**h**) cells treated as in (**a**) (n=3). Scale bar, 100 μm. ***P* < 0.01. Data shown are mean ± SD.

Figure S6

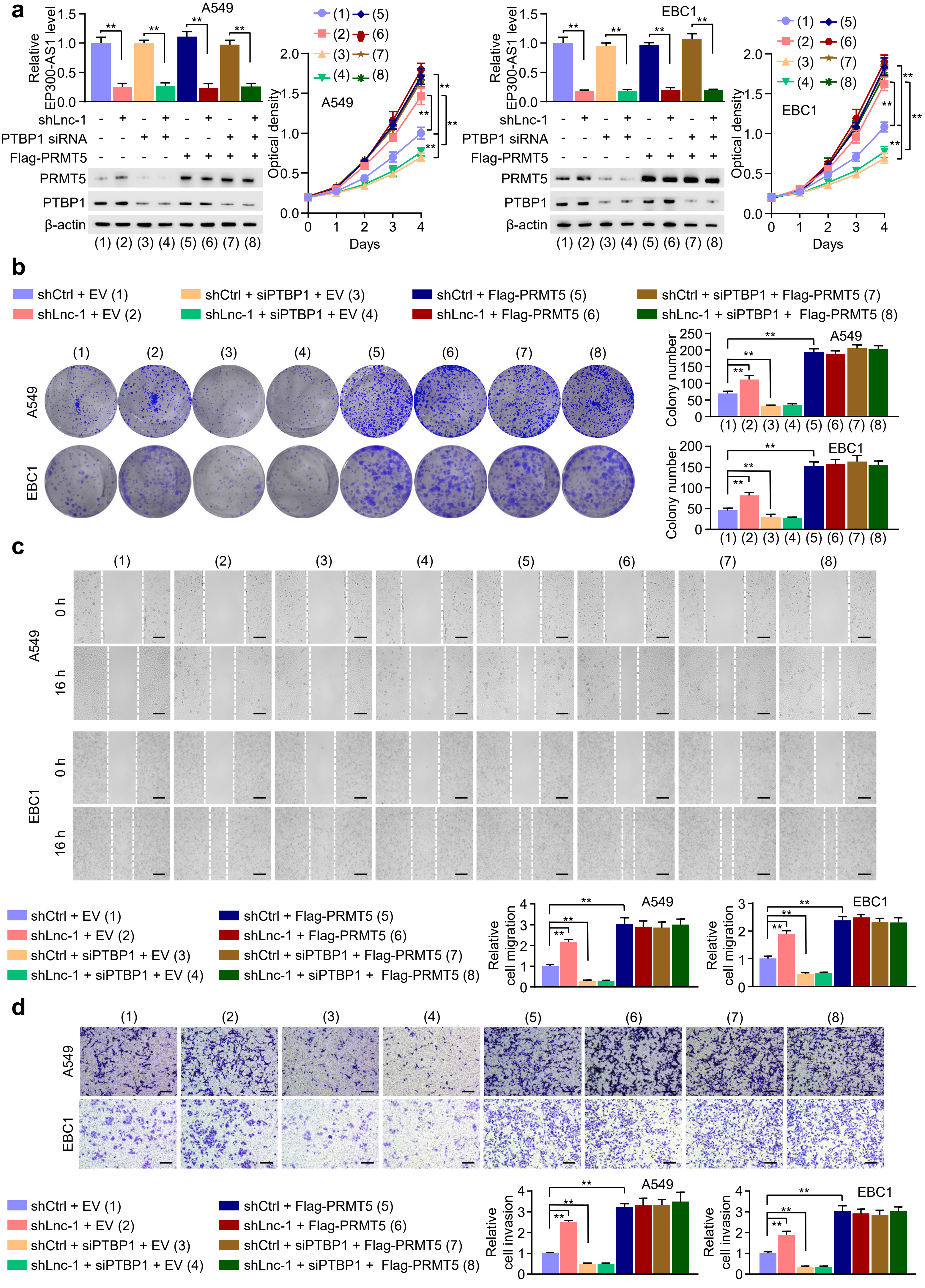

a
A549
EBC1
Relative EP300-AS1 level
shLnc-1
PTBP1 siRNA
Flag-PRMT5
PRMT5
PTBP1
β-actin
(1) (2) (3) (4) (5) (6) (7) (8)
Optical density
Days
b
shCtrl + EV (1)
shLnc-1 + EV (2)
shCtrl + siPTBP1 + EV (3)
shLnc-1 + siPTBP1 + EV (4)
shCtrl + Flag-PRMT5 (5)
shLnc-1 + Flag-PRMT5 (6)
shCtrl + siPTBP1 + Flag-PRMT5 (7)
shLnc-1 + siPTBP1 + Flag-PRMT5 (8)
Colony number
c
0 h
16 h
Relative cell migration
d
Relative cell invasion

Supplementary Fig.6 EP300-AS1-PTBP1-PRMT5 axis regulates NSCLC cell proliferation, migration and invasion

a CCK8 assays for A549 and EBC1 cells stably expressing shCtrl or shLnc-1 and co-transfected with control siRNA or PTBP1 siRNA and empty vector or FLAG-PRMT5. EP300-AS1 expression was examined by qRT-PCR. PRMT5 and PTBP1 expression was examined by Western Blot (n=3). $^{**}P < 0.01$. **b** Colony formation assays for A549 and EBC1 cells treated as in (**a**) (n=3). $^{**}P < 0.01$. **c** Wound-healing assays for A549 and EBC1 cells treated as in (**a**) (n=3). Scale bar, 100 μm. $^{**}P < 0.01$. **d** Transwell assays for A549 and EBC1 cells treated as in (**a**) (n=3). Scale bar, 100 μm. $^{**}P < 0.01$. Data shown are mean ± SD.

Figure S7

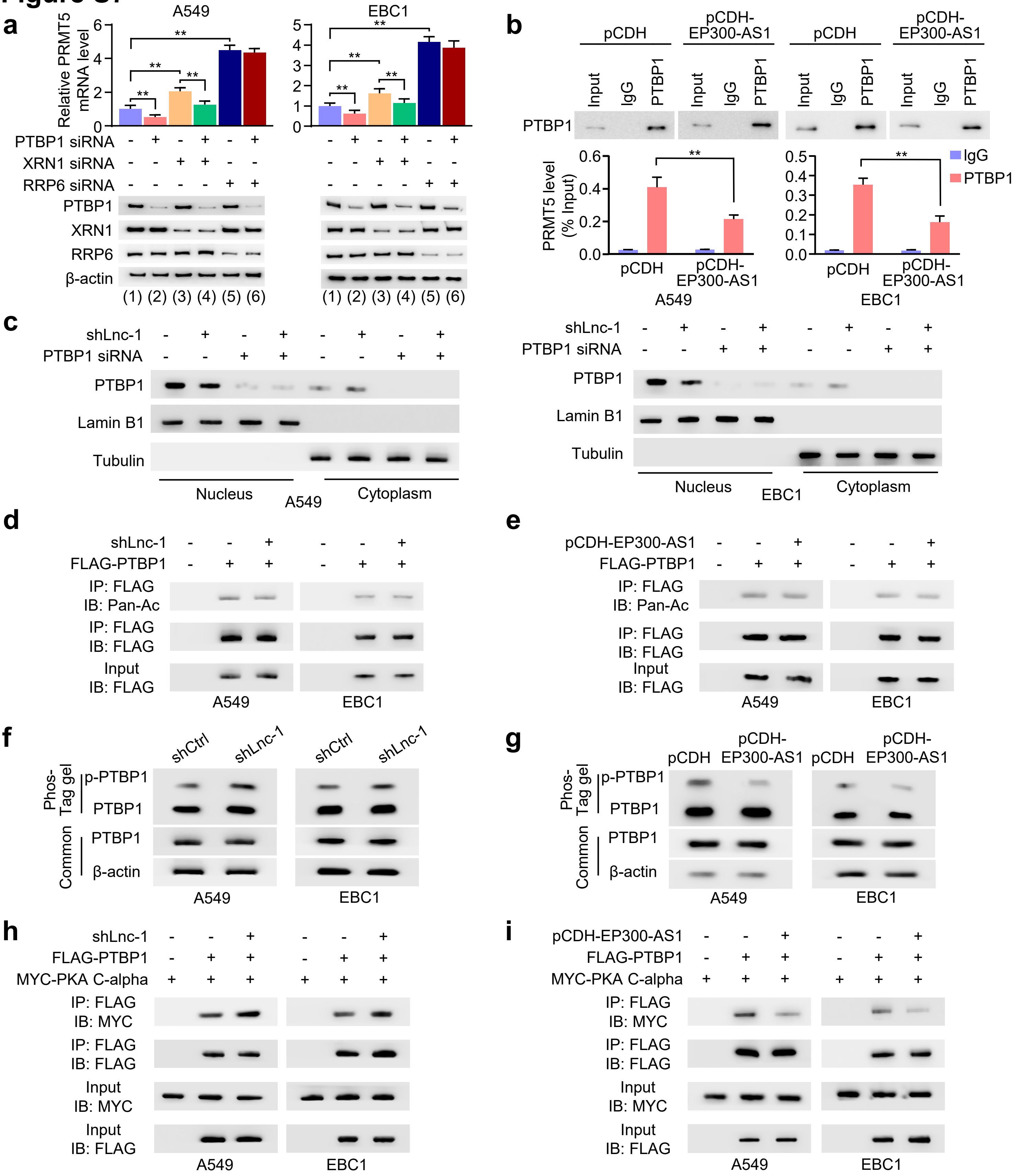

Supplementary Fig.7 EP300-AS1 inbihits PKA-mediated PTBP1 phosphorylation

a A549 and EBC1 cells were transfected with the indicated siRNA and followed by IB and qRT-PCR (n=3). **P < 0.01. **b** RIP assays were performed using antibodies against PTBP1 in A549 and EBC1 cells stably expressing pCDH or pCDH-EP300-AS1 (n=3). **c** IB assay of nuclear and cytoplasmic PTBP1 protein in A549 and EBC1 cells stably expressing shCtrl or shLnc-1 and transfected with control siRNA or PTBP1 siRNA (n=3). **d** A549 and EBC1 cells stably expressing shCtrl or shLnc-1 were transfected with FLAG-PTBP1. Cell lysates were immunoprecipitated with anti-FLAG, followed by IB with pan-acetylation antibodies (n=3). **e** A549 and EBC1 cells stably expressing empty pCDH vector or pCDH-EP300-AS1 were transfected with FLAG-PTBP1, and analyzed as in (**d**) (n=3). **f** Endogenous PTBP1 phosphorylation in A549 and EBC1 cells stably expressing shCtrl or shLnc-1 was analyzed using Phos-Tag gel (n=3). **g** Endogenous PTBP1 phosphorylation in A549 and EBC1 cells stably expressing empty pCDH vector or pCDH-EP300-AS1 was analyzed using Phos-Tag gel (n=3). **h** A549 and EBC1 cells stably expressing shCtrl or shLnc-1 were co-transfected with FLAG-PTBP1 and MYC-PKA C-alpha. Cell lysates were immunoprecipitated with anti-FLAG, followed by IB with the indicated antibodies (n=3). **i** A549 and EBC1 cells stably expressing empty pCDH vector or pCDH-EP300-AS1 were co-transfected with FLAG-PTBP1 and MYC-PKA C-alpha, and analyzed as in (**h**) (n=3). Data shown are mean ± SD.

Figure S8

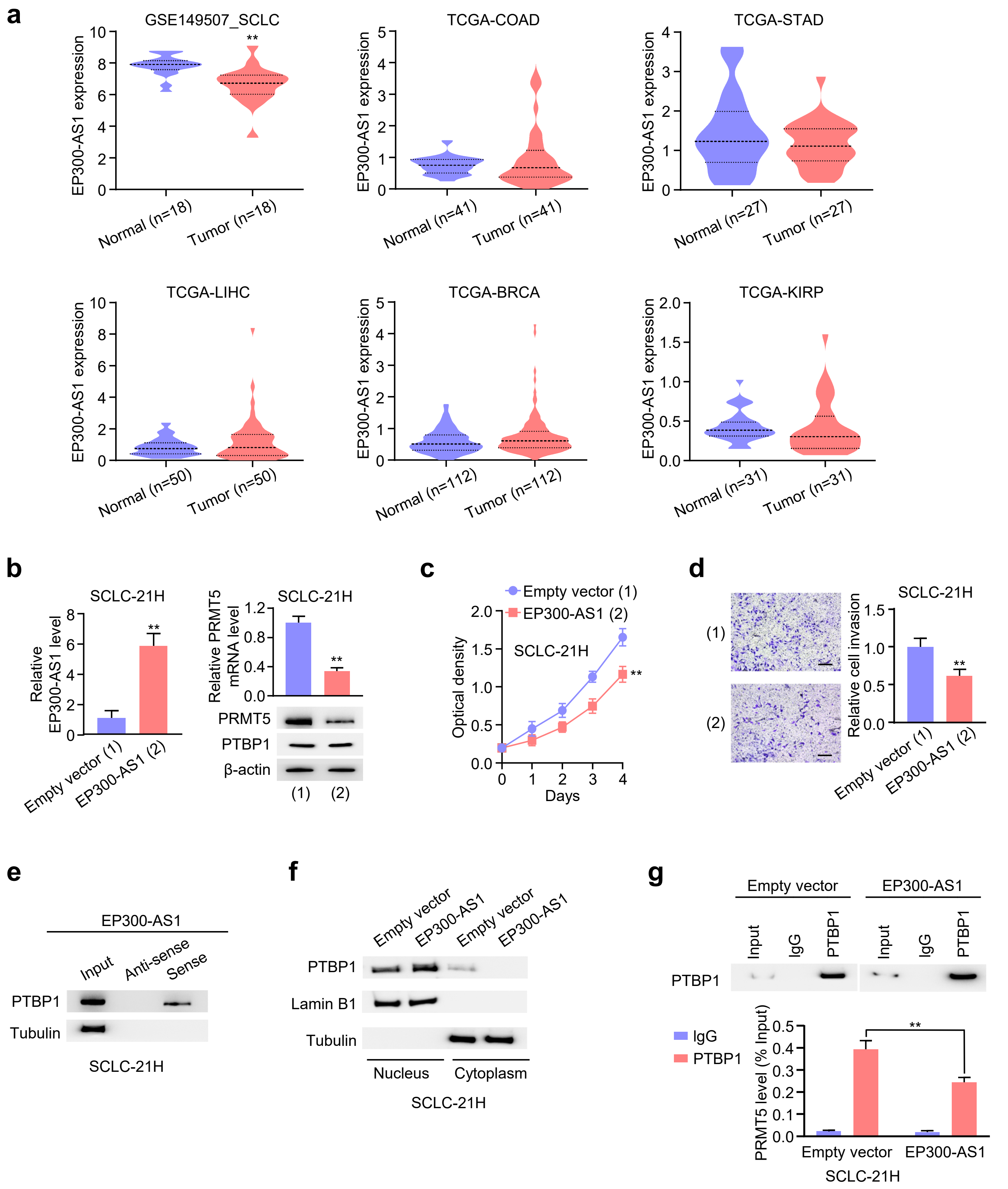

Supplementary Fig.8 Clinical significance of EP300-AS1 in SCLC patients

a The EP300-AS1 expression were compared between various cancerous tissues and their normal tissues in the GSE149507 database or in the Cancer Genome Atlas (TCGA) database. ***P* < 0.01. **b** SCLC-21H cells were transfected with empty vector or EP300-AS1 and followed by qRT-PCR and IB (n=3). ***P* < 0.01. **c, d** CCK8 assays (**c**) and transwell assays (**d**) for SCLC-21H cells infected as in (**b**) (n=3). Scale bar, 100 μm. ***P* < 0.01. **e** Intracellular F2-RNA pulldown assays were performed using the sense and antisense of EP300-AS1, followed by immunoblot (IB) (n=3). **f** IB assay of nuclear and cytoplasmic PTBP1 protein in SCLC-21H expressing empty vector or EP300-AS1 (n=3). **g** RIP assays were performed using antibodies against PTBP1 in SCLC-21H cells expressing empty vector or EP300-AS1 (n=3). ***P* < 0.01. Data shown are mean ± SD.

Figure S9

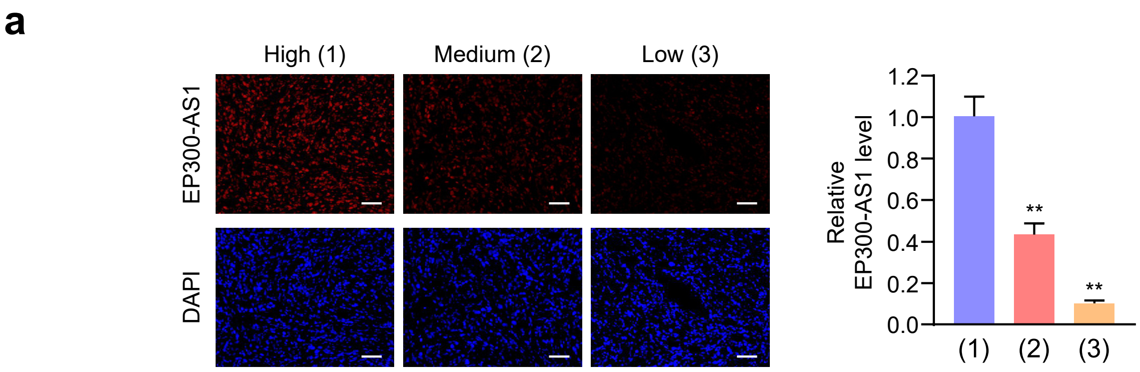

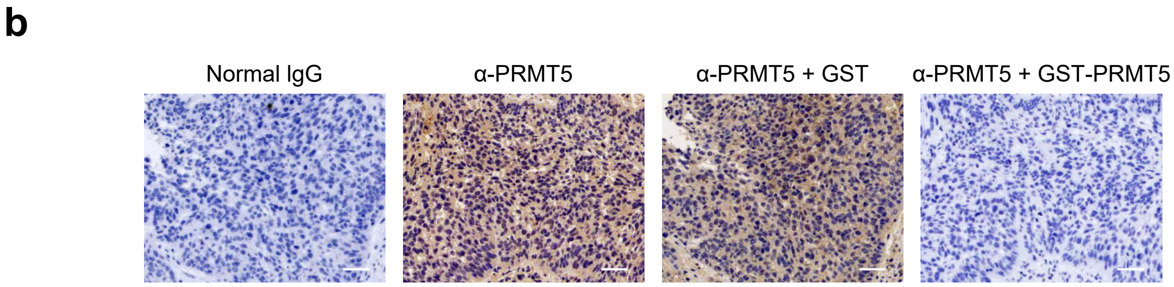

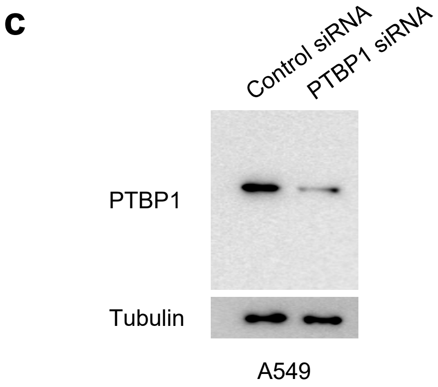

Supplementary Fig.9 Confirmation of the specificity of EP300-AS1 specific fluorescent probe and antibodies

a Different EP300-AS1 expression levels in 3 different human LUAD tissues were examined by FISH and qRT-PCR (n=3). Scale bar, 50 μm. $^{**}P < 0.01$. **b** Human LUAD specimens were incubated with normal IgG or anti-PRMT5. To validate antibody specificity, the anti-ASCT2 was pre-incubated with GST-PRMT5 or GST protein for 1 h prior to applying to tissue. Scale bar, 50 μm. **c** A549 cells were transfected with control siRNA or PTBP1 siRNA, followed by IB with anti-PTBP1. Data shown are mean ± SD.
