# Supplementary material for: LncRNA EP300-AS1 interacts with PTBP1 to destabilize PRMT5 mRNA and suppresses NSCLC growth and metastasis
Source: Cell Death Dis. 2025 Aug 11;16(1):607. doi: 10.1038/s41419-025-07931-3 (PMC12339964; doi:10.1038/s41419-025-07931-3)
Supplement: Supplementary file 2 — Supplementary Table1-3 [file 41419_2025_7931_MOESM2_ESM.docx]

**Table S1. Characteristics of 97 NSCLC patients.**

| No. | Gender | Age | Pathological subtypes | TNM | Stage |
| --- | --- | --- | --- | --- | --- |
| 1  2  3  4  5  6  7  8  9  10  11  12  13  14  15  16  17  18  19  20  21  22  23  24  25  26  27  28  29  30  31  32  33  34  35  36  37  38  39  40  41  42  43  44  45  46  47  48  49  50  51  52  53  54  55  56  57  58  59  60  61  62  63  64  65  66  67  68  69  70  71  72  73  74  75  76  77  78  79  80  81  82  83  84  85  86  87  88  89  90  91  92  93  94  95  96  97 | Female  Female  Female  Female  Female  Female  Male  Female  Female  Female  Female  Female  Male  Female  Male  Female  Male  Male  Male  Female  Female  Male  Female  Female  Female  Male  Female  Female  Female  Female  Female  Female  Female  Female  Female  Female  Female  Female  Female  Female  Male  Male  Female  Female  Female  Female  Male  Female  Female  Female  Male  Male  Male  Male  Male  Male  Male  Male  Male  Male  Male  Male  Male  Male  Male  Male  Male  Male  Male  Male  Male  Male  Male  Male  Male  Male  Male  Male  Male  Male  Male  Male  Male  Male  Male  Male  Male  Male  Male  Male  Male  Male  Male  Male  Male  Male  Male | 50  77  72  77  69  75  66  67  45  72  50  58  73  59  52  62  67  54  71  68  69  58  46  72  64  67  68  70  60  74  54  55  70  65  60  59  59  84  60  71  72  72  58  60  62  71  68  66  59  61  66  54  70  65  59  68  68  61  59  57  69  70  72  72  66  61  77  82  72  57  59  75  71  64  68  52  59  78  66  60  57  60  72  57  76  67  64  72  65  77  68  72  72  68  55  64  71 | LUAD  LUAD  LUAD  LUAD  LUAD  LUAD  LUAD  LUAD  LUAD  LUAD  LUAD  LUAD  LUAD  LUAD  LUAD  LUAD  LUAD  LUAD  LUAD  LUAD  LUAD  LUAD  LUAD  LUAD  LUAD  LUAD  LUAD  LUAD  LUAD  LUAD  LUAD  LUAD  LUAD  LUAD  LUAD  LUAD  LUAD  LUAD  LUAD  LUAD  LUAD  LUAD  LUAD  LUAD  LUAD  LUAD  LUAD  LUAD  LUAD  LUAD  LUAD  LUAD  LUSC  LUSC  LUSC  LUSC  LUSC  LUSC  LUSC  LUSC  LUSC  LUSC  LUSC  LUSC  LUSC  LUSC  LUSC  LUSC  LUSC  LUSC  LUSC  LUSC  LUSC  LUSC  LUSC  LUSC  LUSC  LUSC  LUSC  LUSC  LUSC  LUSC  LUSC  LUSC  LUSC  LUSC  LUSC  LUSC  LUSC  LUSC  LUSC  LUSC  LUSC  LUSC  LUSC  LUSC  LUSC | \| T1bN0M0 \| \| --- \| \| T1cN2bM0 \| \| T3N0M0 \| \| T1cN2aM0 \| \| T1bN1M0 \| \| T1bN1M0 \| \| T2aN0M0 \| \| T1aN0M0 \| \| T1bN0M0 \| \| T1bN0M0 \| \| T1bN1M0 \| \| T1cN2bM0 \| \| T2aN0M0 \| \| T1bN1M0 \| \| T1cN0M0 \| \| T3N0M0 \| \| T1cN2bM0 \| \| T1bN2bM0 \| \| T1cN0M0 \| \| T2aN0M0 \| \| T1bN0M0 \| \| T1bN0M0 \| \| T1cN2bM0 \| \| T2aN0M0 \| \| T1bN2bM0 \| \| T1bN0M0 \| \| T2aN0M0 \| \| T2aN0M0 \| \| T1bN0M0 \| \| T1cN0M0 \| \| T1cN2bM0 \| \| T1bN0M0 \| \| T2aN0M0 \| \| T1cN0M0 \| \| T1bN0M0 \| \| T1bN0M0 \| \| T1bN0M0 \| \| T1bN1M0 \| \| T1cN0M0 \| \| T2aN0M0 \| \| T1cN0M0 \| \| T1aN0M0 \| \| T2aN0M0 \| \| T1aN0M0 \| \| T1cN0M0 \| \| T2aN0M0 \| \| T1cN0M0 \| \| T1bN1M0 \| \| T1bN0M0 \| \| T1bN0M0 \| \| T1cN0M0 \| \| T1bN0M0 \| \| T1cN0M0 \| \| T1bN1M0 \| \| T3N0M0 \| \| T1bN2bM0 \| \| T1cN0M0 \| \| T2aN2aM0 \| \| T3N0M0 \| \| T1cN0M0 \| \| T2aN2bM0 \| \| T1bN2bM0 \| \| T1cN0M0 \| \| T2aN0M0 \| \| T1bN2bM0 \| \| T4N0M0 \| \| T4N0M0 \| \| T3N0M0 \| \| T2aN0M0 \| \| T4N0M0 \| \| T1cN0M0 \| \| T1bN2bM0 \| \| T1bN1M0 \| \| T2aN0M0 \| \| T1cN0M0 \| \| T2aN0M0 \| \| T1cN0M0 \| \| T2aN0M0 \| \| T1cN0M0 \| \| T4N0M0 \| \| T1bN1M0 \| \| T3N0M0 \| \| T2aN0M0 \| \| T1bN0M0 \| \| T1cN2bM0 \| \| T1cN0M0 \| \| T1bN0M0 \| \| T2aN2bM0 \| \| T2aN0M0 \| \| T4N0M0 \| \| T2bN0M0 \| \| T1cN0M0 \| \| T1cN0M0 \| \| T2aN0M0 \| \| T1cN0M0 \| \| T1cN0M0 \| \| T2aN0M0 \| | IA2  IIIA  IIB  IIB  IIA  IIA  IB  IA1  IA2  IA2  IIA  IIIA  IB  IIA  IA3  IIB  IIIA  IIIA  IA3  IB  IA2  IA2  IIIA  IB  IIIA  IA2  IB  IB  IA2  IA3  IIIA  IA2  IB  IA3  IA2  IA2  IA2  IIA  IA3  IB  IA3  IA1  IB  IA1  IA3  IB  IA3  IIA  IA2  IA2  IA3  IA2  IA3  IIA  IIB  IIIA  IA3  IIIA  IIB  IA3  IIIB  IIIA  IA3  IB  IIIA  IIIA  IIIA  IIB  IB  IIIA  IA3  IIIA  IIA  IB  IA3  IB  IA3  IB  IA3  IIIA  IIA  IIB  IB  IA2  IIIA  IA3  IA2  IIIB  IB  IIIA  IIA  IA3  IA3  IB  IA3  IA3  IB |

**Table S2. The cDNA target sequences of shRNAs or siRNAs.**

| Gene | Target sequence (5’ → 3’) |
| --- | --- |
| PTBP1 (siRNA) | GCACAGTGTTGAAGATCAT |
| XRN1 (siRNA) | AGAUGAACUUACCGUAGAA |
| RRP6 (siRNA) | GAAGGCAGCUGAGCAAACA |
| EP300-AS1 (shRNA) (shLnc-1) | GGTCCACCTCCTAACAAAT |
| EP300-AS1 (shRNA) (shLnc-2) | GAGATGGAGGCTTACTCTA |

**Table S3. Primers used for quantitative real-time PCR.**

| Gene | Species | Forward (5’ → 3’) | Reverse (5’ → 3’) |
| --- | --- | --- | --- |
| EP300-AS1 | Human | TGCGGGCGTCCTACCAGTG | CCCTCCTCCACAGCCGTCAC |
| PRMT5 | Human | CTGTCTTCCATCCGCGTTTCA | GCAGTAGGTCTGATCGTGTCTG |
| α-Tubulin | Human | CCAAGCTGGAGTTCTCTA | CAATCAGAGTGCTCCAGG |
