# Supplementary material for: LncRNA EP300-AS1 interacts with PTBP1 to destabilize PRMT5 mRNA and suppresses NSCLC growth and metastasis
Source: Cell Death Dis. 2025 Aug 11;16(1):607. doi: 10.1038/s41419-025-07931-3 (PMC12339964; doi:10.1038/s41419-025-07931-3)

Fig.3a

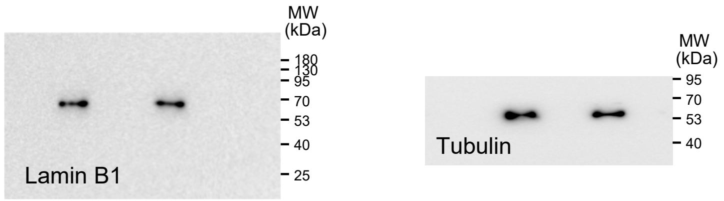

Fig.3e

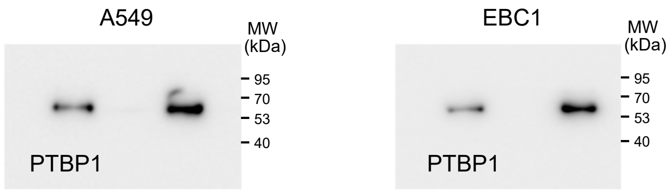

Fig.3f

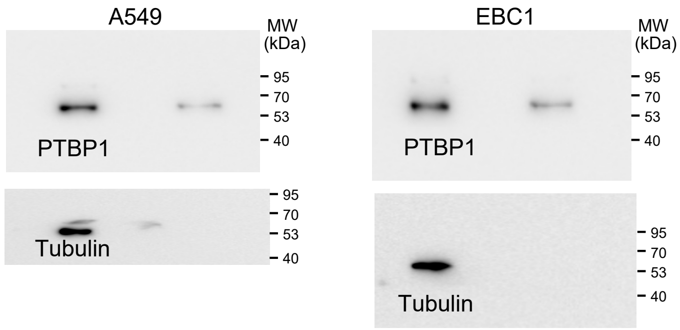

Fig.3g

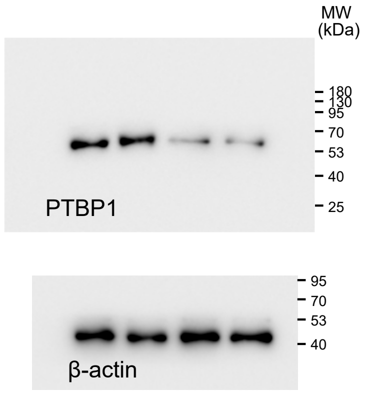

Fig.4c

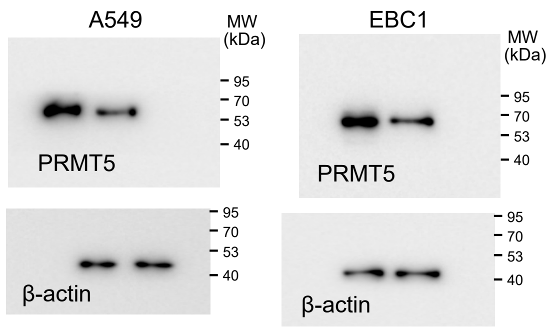

Fig.4d

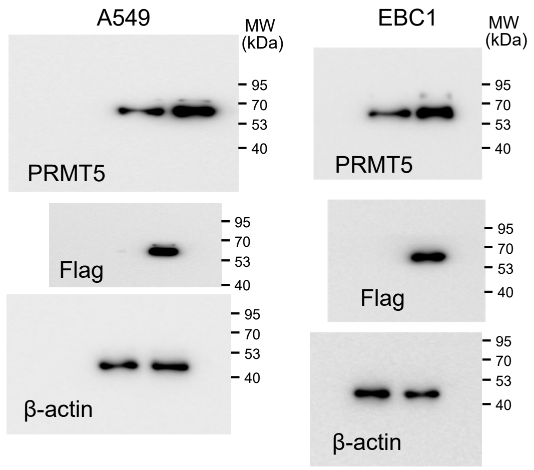

Fig.4g

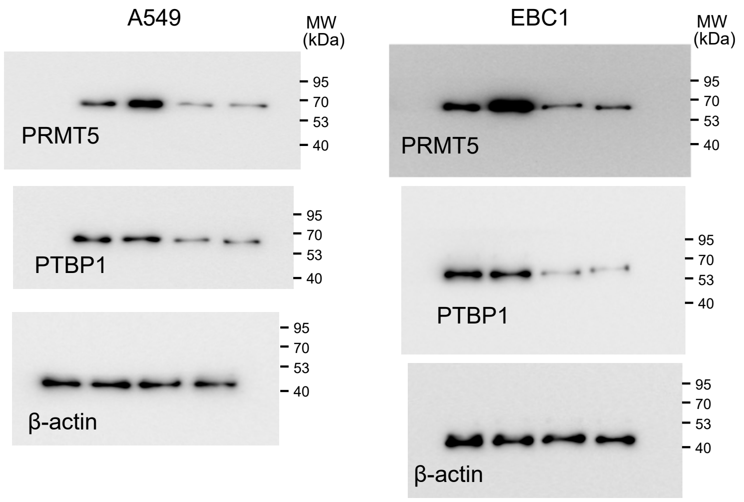

Fig.4i

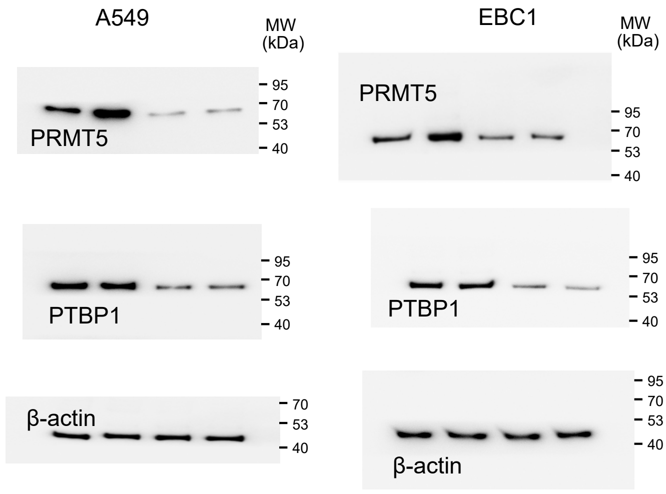

Fig.5a

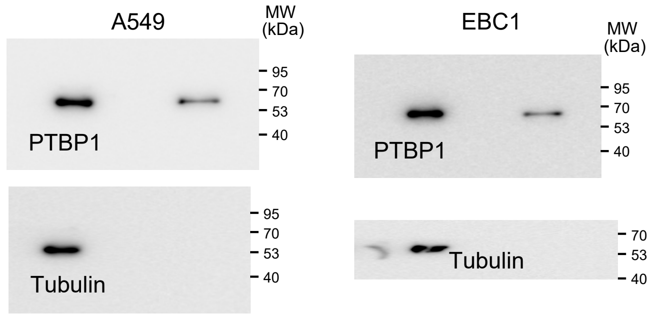

Fig.5c

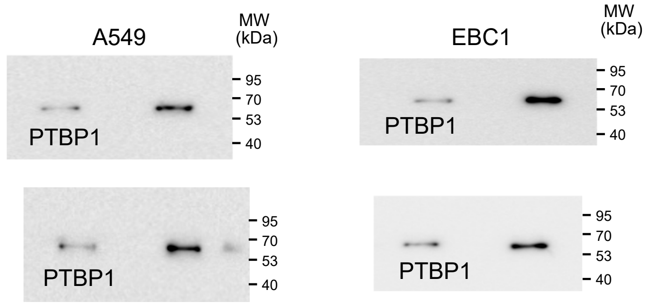

Fig.5e

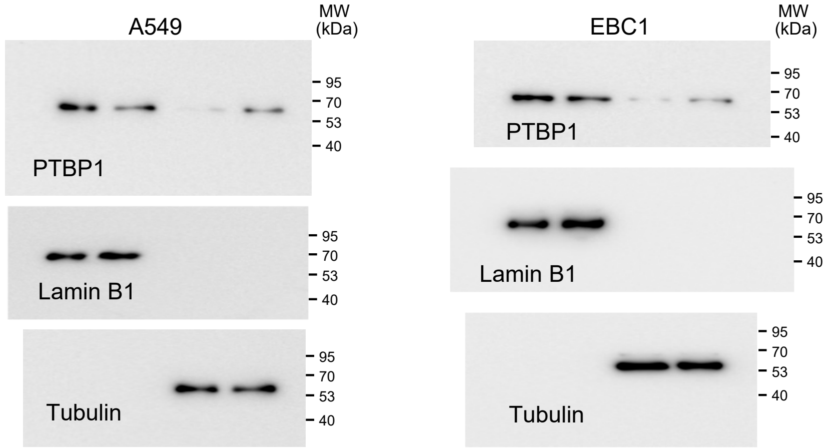

Fig.5f

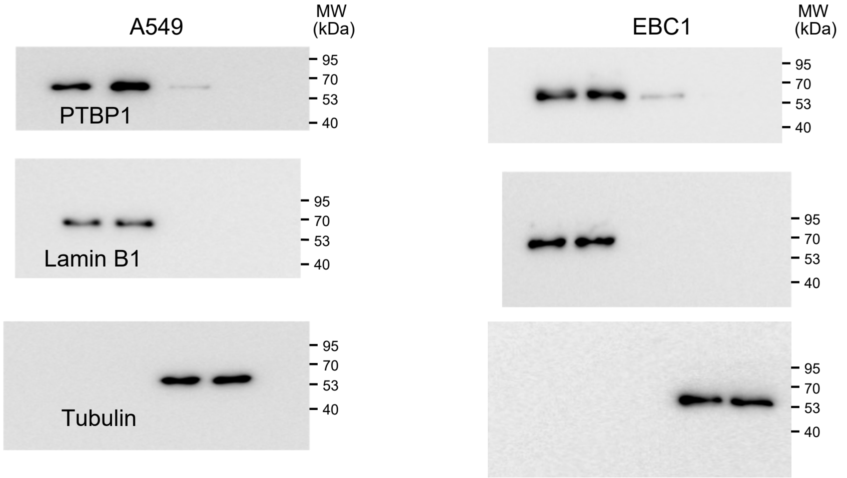

Fig.S3a

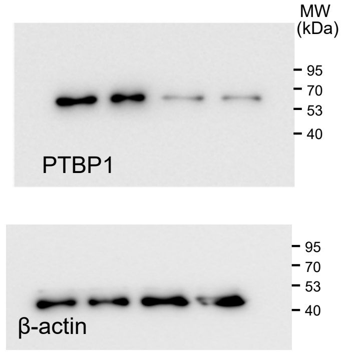

Fig.S4a

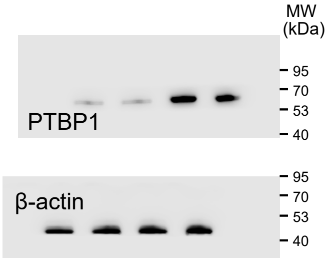

Fig.S4b

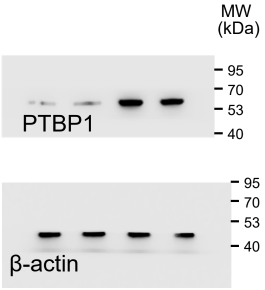

Fig.S6a

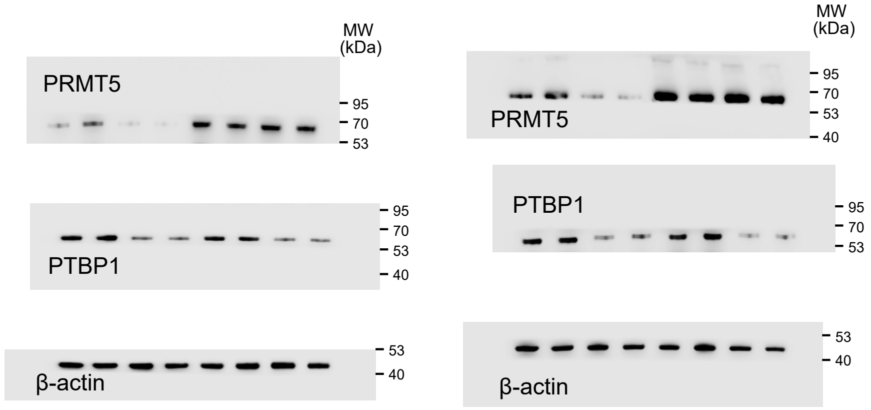

Fig.S7a

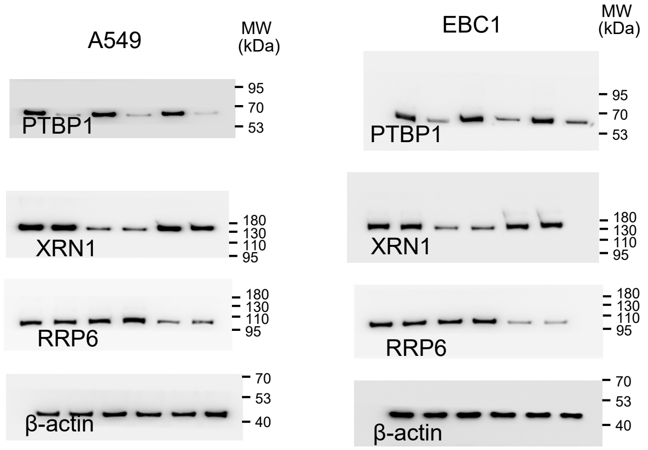

Fig.S7b

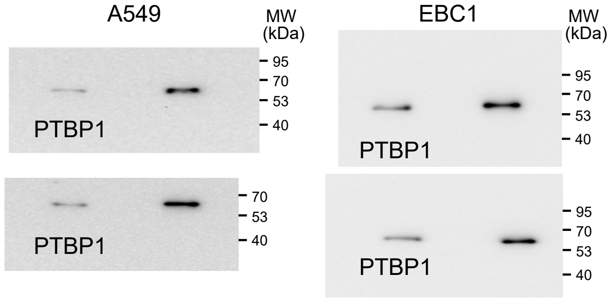

Fig.S7c

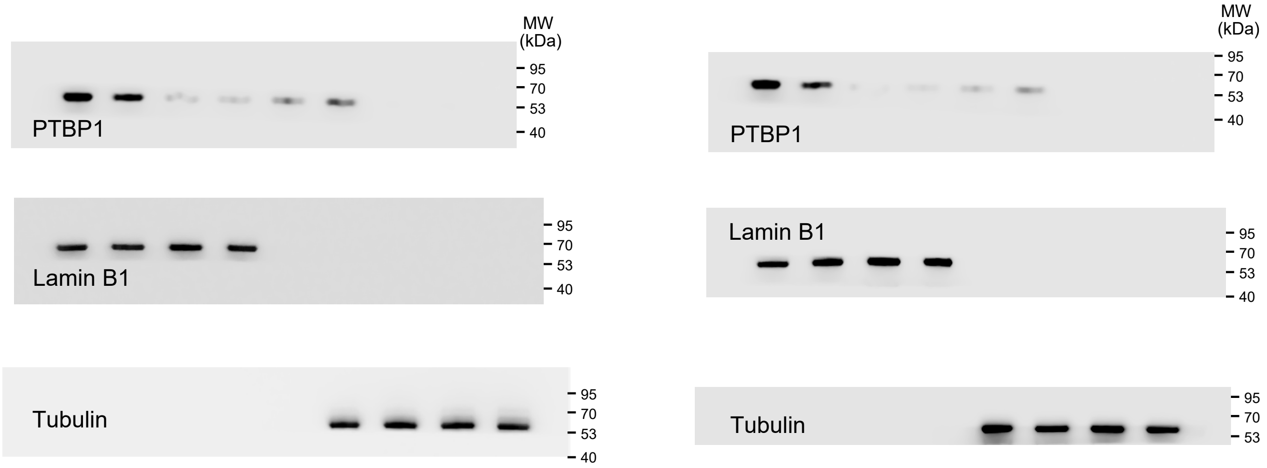

Fig.S7d

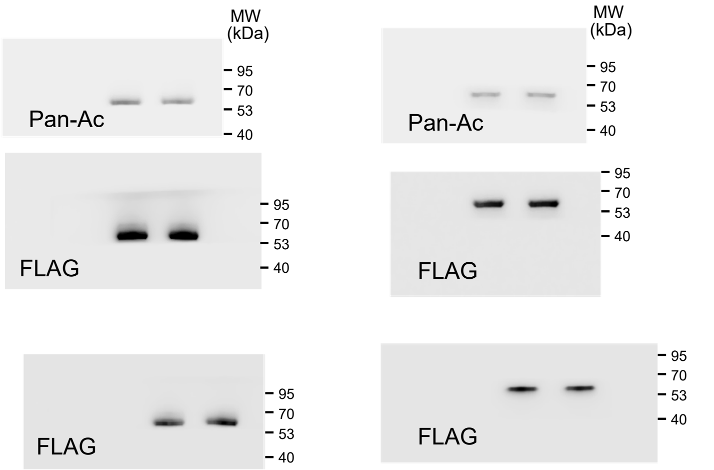

Fig.S7e

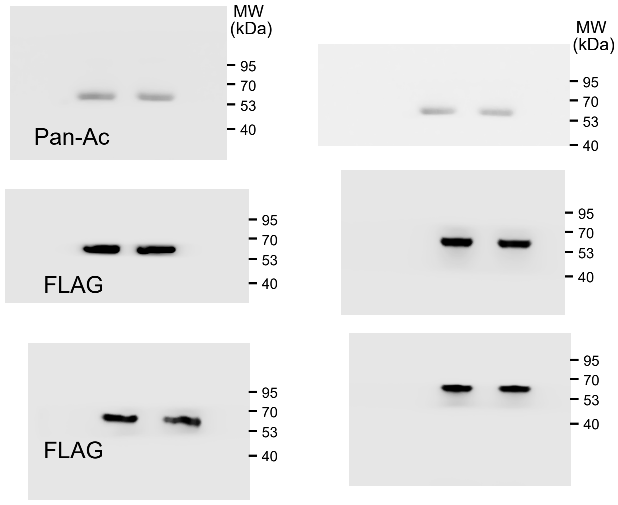

Fig.S7f

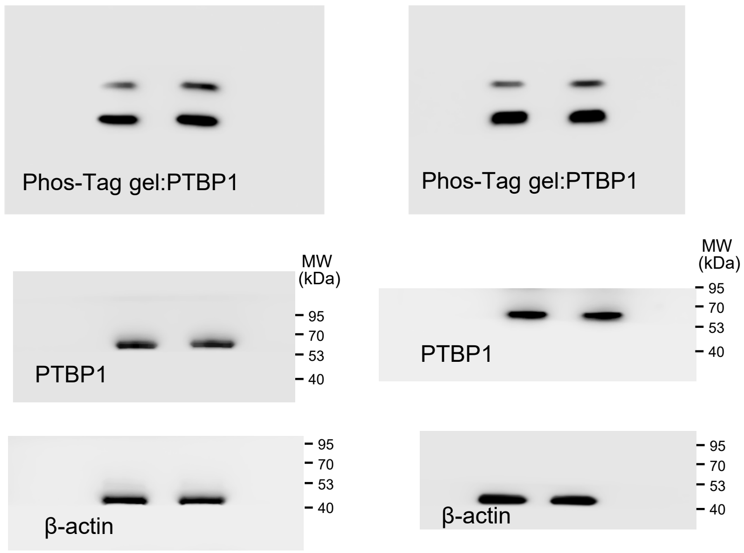

Fig.S7g

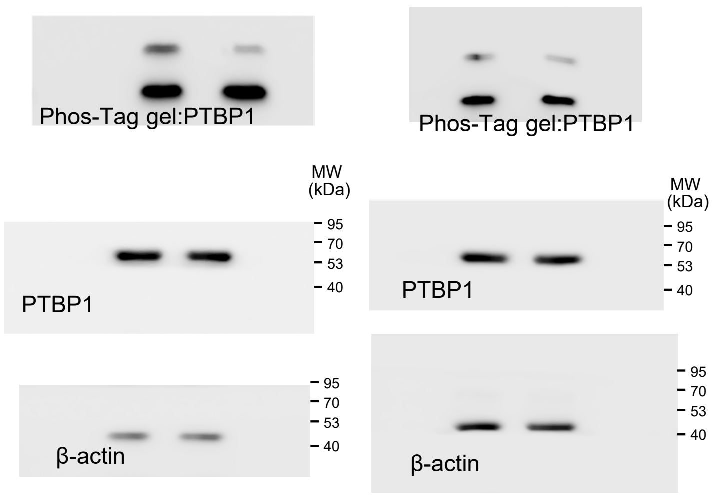

Fig.S7h

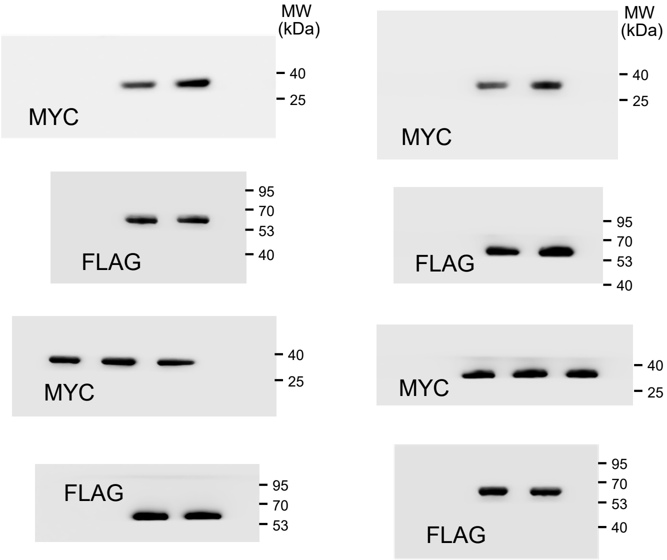

Fig.S7i

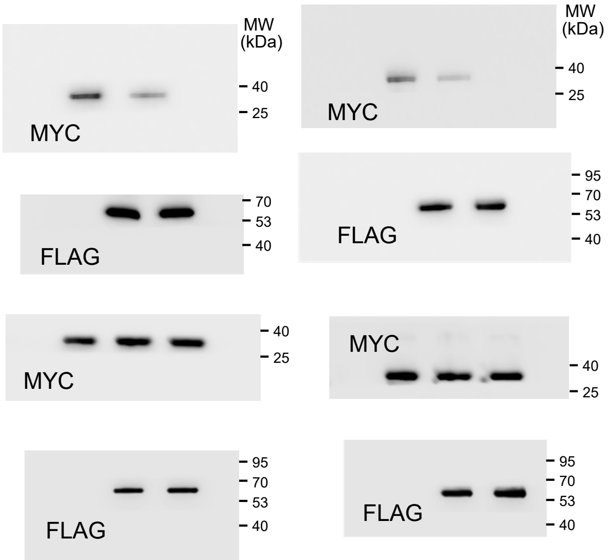

Fig.S8b

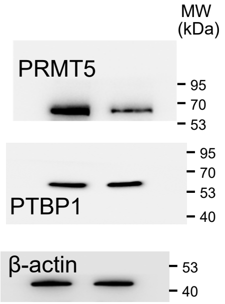

Fig.S8e

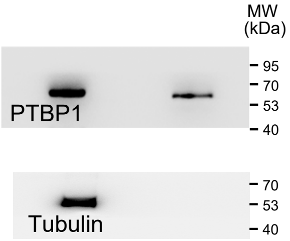

Fig.S8f

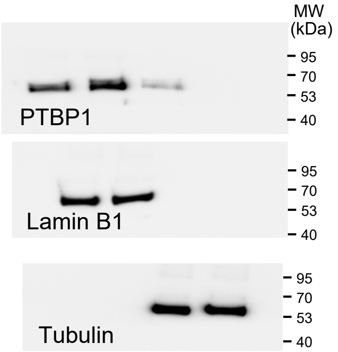

Fig.S8g

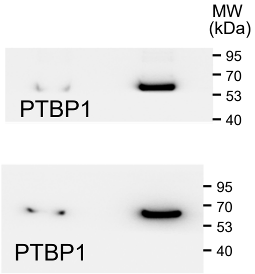

Fig.S9c

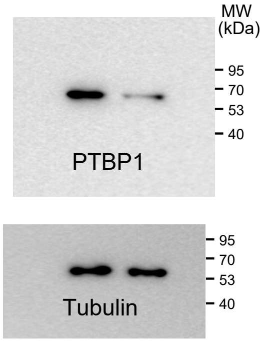

Supplement: Supplementary file 3 — Original data files [file 41419_2025_7931_MOESM3_ESM.pdf]
